# FTO fuels diabetes-induced vascular endothelial dysfunction associated with inflammation by erasing m^6^A methylation of *TNIP1*

**DOI:** 10.1172/JCI160517

**Published:** 2023-10-02

**Authors:** Chuandi Zhou, Xinping She, Chufeng Gu, Yanan Hu, Mingming Ma, Qinghua Qiu, Tao Sun, Xun Xu, Haibing Chen, Zhi Zheng

**Affiliations:** 1 Department of Ophthalmology, Shanghai General Hospital, Shanghai Jiao Tong University School of Medicine, National Clinical Research Center for Eye Diseases, Shanghai Key Laboratory of Ocular Fundus Diseases, Shanghai Engineering Center for Visual Science and Photomedicine, Shanghai Engineering Center for Precise Diagnosis and Treatment of Eye Diseases, Shanghai, China.; 2 Shanghai Eye Diseases Prevention and Treatment Center, Shanghai Eye Hospital, Shanghai General Hospital, National Clinical Research Center for Eye Diseases, Shanghai Key Laboratory of Ocular Fundus Diseases, Shanghai, China.; 3 Department of Endocrinology and Metabolism, Shanghai 10th People’s Hospital, School of Medicine, Tongji University, Shanghai, China.

**Keywords:** Inflammation, Vascular Biology, Diabetes, Endothelial cells, Epigenetics

## Abstract

Endothelial dysfunction is a critical and initiating factor of the vascular complications of diabetes. Inflammation plays an important role in endothelial dysfunction regulated by epigenetic modifications. N6-methyladenosine (m^6^A) is one of the most prevalent epigenetic modifications in eukaryotic cells. In this research, we identified an m^6^A demethylase, fat mass and obesity-associated protein (FTO), as an essential epitranscriptomic regulator in diabetes-induced vascular endothelial dysfunction. We showed that enhanced FTO reduced the global level of m^6^A in hyperglycemia. FTO knockdown in endothelial cells (ECs) resulted in less inflammation and compromised ability of migration and tube formation. Compared with EC *Fto*^fl/fl^ diabetic mice, EC-specific *Fto*-deficient (EC *Fto*^Δ/Δ^) diabetic mice displayed less retinal vascular leakage and acellular capillary formation. Furthermore, methylated RNA immunoprecipitation sequencing (MeRIP-Seq) combined with RNA-Seq indicated that *Tnip1* served as a downstream target of FTO. Luciferase activity assays and RNA pull-down demonstrated that FTO repressed *TNIP1* mRNA expression by erasing its m^6^A methylation. In addition, TNIP1 depletion activated NF-κB and other inflammatory factors, which aggravated retinal vascular leakage and acellular capillary formation, while sustained expression of *Tnip1* by intravitreal injection of adeno-associated virus alleviated endothelial impairments. These findings suggest that the FTO-TNIP1-NF-κB network provides potential targets to treat diabetic vascular complications.

## Graphical abstract

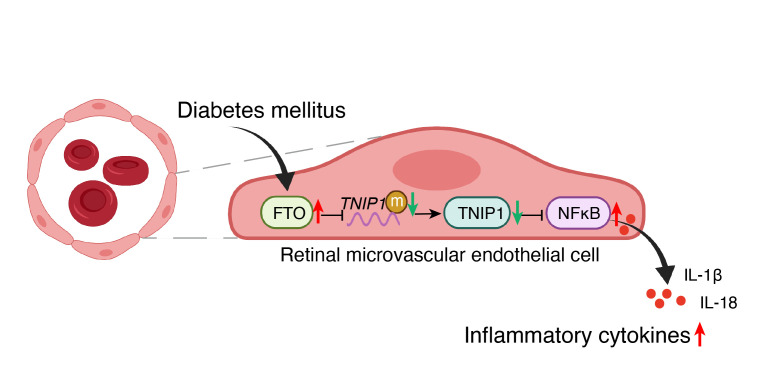

## Introduction

Diabetes mellitus is a global epidemic affecting approximately 10% of the world population (1). Despite advances in normalizing glucose homeostasis, the prevention and treatment of diabetic vascular complications remain a major challenge (2, 3). Accumulating evidence indicates that endothelial dysfunction is the critical and initiating factor of micro- and macrovascular complications (4, 5). In people with diabetes, the prevalence of diabetes-induced endothelial dysfunction was estimated to be 18.8% (microvascular) and 12.7% (macrovascular) globally in 2018. Endothelial dysfunction causes major damage that leads to microvascular complications, including retinopathy, nephropathy, and neuropathy, as well as macrovascular complications manifested as coronary artery disease, heart failure, and stroke. In addition, it is well established that inflammation plays a critical role in the pathogenesis of diabetes-associated vascular endothelial dysfunction (7, 8). However, the mechanisms underlying vascular endothelial dysfunction induced by inflammation in diabetes remain largely unknown.

Epigenetic modifications in diabetes and its associated complications have been extensively investigated. Previous studies mainly focused on DNA methylation, histone modifications, and noncoding RNAs (9, 10). Our group has demonstrated that the class III histone deacetylase sirtuin 1 (SIRT1) functioned as a target of miR-23b-3p regulating the inflammation of vascular endothelial cell (EC) in diabetic retinopathy (11, 12). Notably, N6-methyladenosine (m^6^A) is the most abundant internal modification of mRNA in mammals (13). m^6^A modification is particularly important for meiosis (14), DNA damage response (15), tumorigenesis (16), neuronal function, and sex determination (17). Initially, m^6^A, regulated by methyltransferases (“writers”), was considered to be a static process. Once fat mass and obesity-associated protein (FTO) was discovered as a demethylase (“eraser”), m6A modification was recognized as a dynamic and reversible regulatory process (18, 19). A case-control study confirmed that the m^6^A level was decreased in the peripheral blood of patients with diabetes; this was attributable to elevated FTO (20). FTO was originally found to be a critical regulator in obesity and metabolism (21). Further studies have shown that endothelial FTO deficiency can protect mice against high-fat diet–induced glucose intolerance and insulin resistance by enhancing AKT phosphorylation (22). Bego et al. reported a significant association between the FTO genetic variant rs8050136 A>C and type 2 diabetes, obesity, and inflammation (23). To date, the fundamental role of FTO as a demethylase in diabetes-induced vascular endothelial dysfunction has not been reported to our knowledge.

In the present study, we elucidated the pathological role of FTO-dependent RNA hypomethylation in retinal endothelial homeostasis. The results revealed aberrant m^6^A depletion, accompanied by excessive FTO in diabetes. Loss of FTO prevented endothelial leakage by stabilizing *TNIP1* and suppressing NF-κB in an m^6^A-dependent manner. This study’s results represent important translational implications that interfering FTO and its associated inflammation pathway might provide a promising therapeutic strategy for the vascular complications of diabetes.

## Results

### Decreased m^6^A and elevated FTO are detected in diabetes-induced retinal vascular endothelial dysfunction.

**Figure 1:**
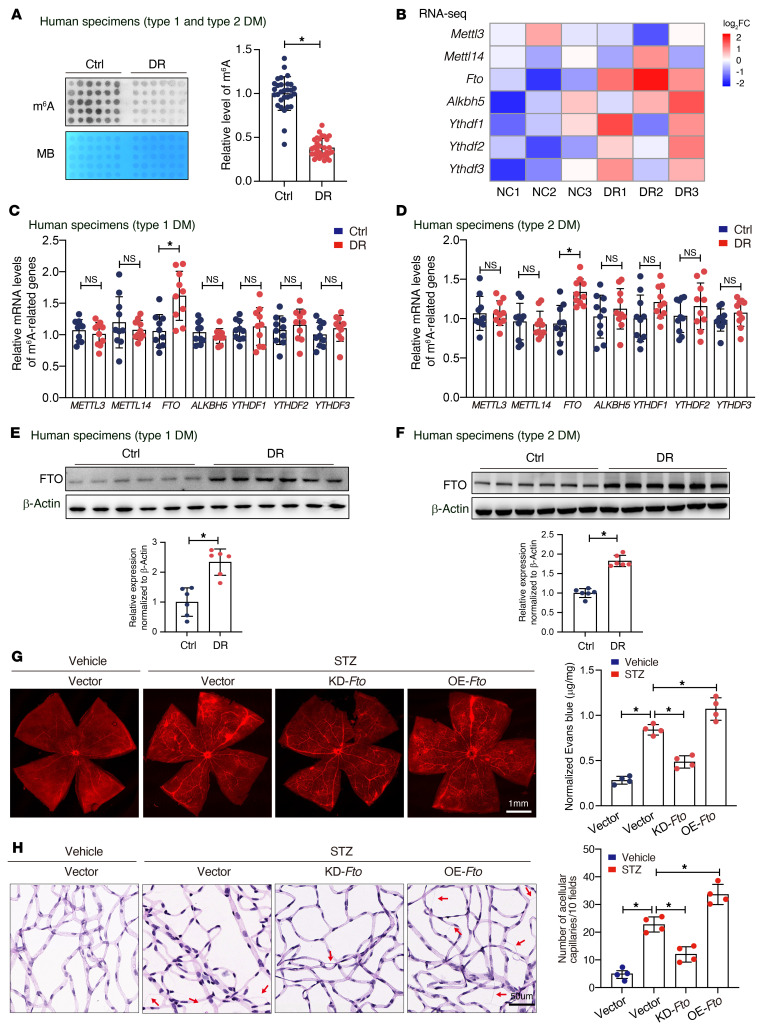
Diabetes induces decreased m^6^A modification and increased FTO expression in human and mice. (**A**) Dot blot showing reduced m^6^A content in the retinal fibrovascular membranes of patients with diabetic retinopathy (DR) (control group, *n* = 30; DR group, type 1 diabetes [left 2 columns], *n* = 10; type 2 diabetes [right 4 columns], *n* = 20; Student’s *t* test). MB, methylene blue staining. (**B**) A heatmap of RNA expression showing an overview of m^6^A-related genes in diabetic retinas. *Fto* was elevated stressed by diabetes (*n* = 3, Mann-Whitney *U* test). (**C** and **D**) qRT-PCR revealed higher levels of *FTO* in retinal fibrovascular membranes of patients with retinopathy due to type 1 (**C**, *n* = 10) or type 2 (**D**, *n* = 10) diabetes (Mann-Whitney *U* test). (**E** and **F**) Western blotting showing elevated expression of FTO in retinal fibrovascular membranes of patients with retinopathy due to type 1 (**E**, *n* = 6) or type 2 (**F**, *n* = 6) diabetes (Student’s *t* test). (**G**) Evans blue dye displayed that silencing *Fto* alleviated diabetes-induced retinal endothelium vascular leakage and enhanced *Fto* aggravated endothelium vascular leakage. A representative image with the quantification of the fluorescence signal is shown (*n* = 4, scale bar: 1 mm). (**H**) Retinal trypsin digestion assays indicate that silencing *Fto* presented with fewer acellular retinal capillaries after the induction of diabetes, and overexpressed *Fto* increased the number of acellular retinal capillaries. Red arrows indicate acellular capillaries. Acellular capillaries are quantified in 20 high-power fields and averaged (*n* = 4, scale bar: 50 μm). For **G** and **H**, significant differences were assessed by Kruskal-Wallis’s test followed by Bonferroni’s post hoc comparison test. Data are shown as the mean ± SD. *P < 0.05.

To investigate the role of m^6^A in diabetes-induced vascular endothelial dysfunction, we first explored the m^6^A level in retinal fibrovascular membranes (Supplemental Figure 1A; supplemental material available online with this article; https://doi.org/10.1172/JCI160517DS1) of the patients with diabetic retinopathy due to type 1 or type 2 diabetes by dot blot assay. Compared with patients with idiopathic epiretinal membrane, m^6^A levels were lower in patients with diabetic retinopathy, irrespective of diabetes type (Figure 1A). Animal models of diabetes were induced by intraperitoneal injection of streptozotocin (STZ), as indicated in Supplemental Figure 1B. RNA-Seq was performed to identify differentially expressed genes in diabetic retinas. A heatmap of RNA expression showed an overview of m^6^A-related genes in diabetic retinas. *Fto* was consistently elevated by diabetes in murine (Figure 1B and Supplemental Figure 1C) retinas. The mRNA levels of m^6^A-related genes, including writers (*METTL3* and *METTL14*), erasers (*FTO* and *ALKBH5*), and readers (m^6^A binding proteins, such as *YTHDF1*, *YTHDF2*, and *YTHDF3*), were assayed by quantitative real-time PCR (qRT-PCR), and the results confirmed that *FTO* was notably elevated in patients with retinopathy due to type 1 (Figure 1C) and type 2 (Figure 1D) diabetes. Parallel findings were also observed in diabetic mice (Supplemental Figure 1, D and E). FTO expression was verified by Western blotting both in vivo and in vitro. The FTO level in retinal fibrovascular membranes increased by nearly 1.8~2.3-fold in patients with diabetes (type 1 diabetes, Figure 1E; type 2 diabetes, Figure 1F). Mice with diabetic retinopathy showed a 1.9~2.1-fold increase in retinal FTO when compared with their wild-type littermates (type 1 diabetes, Supplemental Figure 1F; type 2 diabetes, Supplemental Figure 1G).

In human retinal microvascular ECs (HRMECs), high glucose treatment upregulated FTO expression (Supplemental Figure 1H) in a dose-dependent manner (Supplemental Figure 1I). Immunofluorescence assays verified this finding that there was a clear trend of higher concentration of glucose with decreasing level of m^6^A and increasing expression of FTO (Supplemental Figure 1J).

To determine the role of FTO in diabetes-induced retinal vascular endothelial dysfunction, we modulated its expression by intravitreal injection of adeno-associated virus (AAV) vectors containing overexpression plasmid (Supplemental Figure 1K) or siRNAs (Supplemental Figure 1L). Immunofluorescence displayed successful overexpression or knockout of *Fto* in retinal microvascular ECs after injection of AAV (Supplemental Figure 1M). It was found that overexpressed *Fto* significantly exacerbate retinal vascular leakage (Figure 1G) and increased the number of acellular capillaries in diabetes (Figure 1H), while *Fto* knockdown attenuated these microvascular injuries. These data suggest that diabetes-induced retinal vascular endothelial dysfunction is characterized by decreased m^6^A modification, possibly through the regulation of enhanced FTO.

### FTO accelerates diabetes-induced retinal vascular endothelial dysfunction.

**Figure 2:**
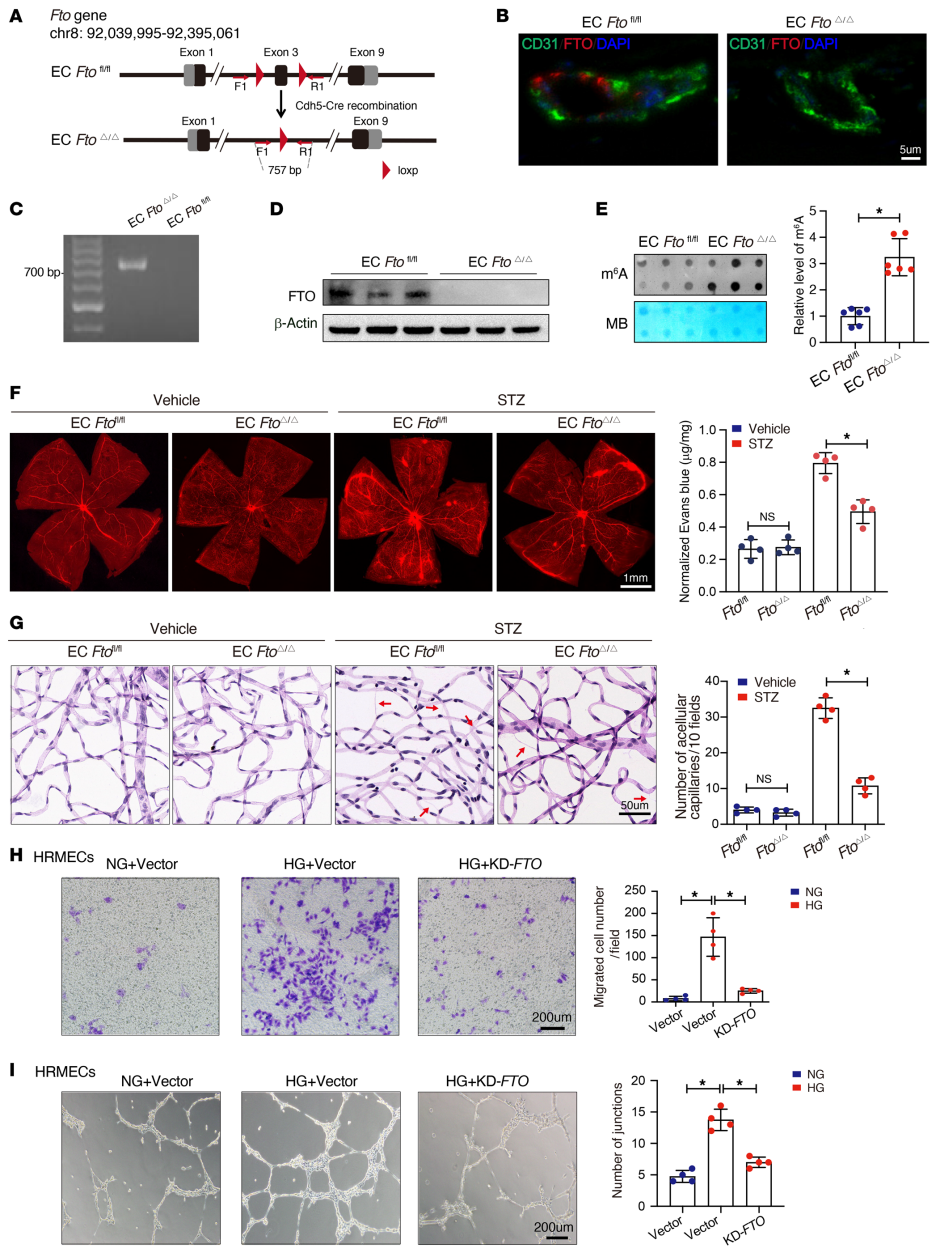
FTO causes retinal vascular endothelial dysfunction in diabetic mice. (**A**) A schematic diagram showing the generation of endothelial cell–specific (EC-specific) *Fto*-deficient (EC *Fto*^Δ/Δ^) mice. (**B**) Immunofluorescence of FTO protein (red), cell nuclei (DAPI, blue), and retinal microvascular ECs (CD31, green) in EC *Fto*^fl/fl^ and EC *Fto*^Δ/Δ^ mice (scale bar: 5 μm). (**C**) PCR genotyping verified *Fto* exon 3 deletion in primary retinal microvascular ECs from EC *Fto*^Δ/Δ^ mice. (**D**) Depletion of FTO protein in the primary retinal microvascular ECs from EC *Fto*^Δ/Δ^ mice (*n* = 3). (**E**) Dot blot showing increased m^6^A content in EC *Fto*^Δ/Δ^ mice (*n* = 6, Student’s *t* test). MB, methylene blue staining. (**F**) Endothelial knockout of *Fto* alleviated diabetes-induced retinal endothelium vascular leakage as shown in flat-mounted retinas stained with Evans blue dye. A representative image with the quantification of the fluorescence signal is shown (*n* = 4, scale bar: 1 mm). (**G**) EC *Fto*^Δ/Δ^ mice presented with fewer acellular retinal capillaries after the induction of diabetes, as indicated by retinal trypsin digestion. Red arrows indicate acellular capillaries. Acellular capillaries were quantified in 20 high-power fields and averaged (*n* = 4, scale bar: 50 μm). (**H**) Transwell assays showing that *FTO* enhances the migration ability of human retinal microvascular ECs (HRMECs) cultivated in high glucose. The number of migrated cells was quantified (*n* = 4, scale bar: 200 μm). (**I**) *FTO* increased tube formation of HRMECs treated with high glucose. The average number of tube formation for each field was assessed (*n* = 4, scale bar: 200 μm). NG, normal glucose (5.5 mM) with D-mannitol as osmotic control; HG, high glucose (30 mM). For **F**–**I**, significant differences were determined by 1-way ANOVA or Kruskal-Wallis’s test followed by Bonferroni’s post hoc comparison test. Data are shown as the mean ± SD. *P < 0.05.

To systematically elucidate the function of FTO in diabetes-induced retinal vascular endothelial dysfunction, EC-specific *Fto*-deficient (EC *Ft*o^Δ/Δ^) mice were generated, as shown in the schematic illustration in Figure 2A. To validate the successful knockout of *Fto*, the mouse retinal vessels were immunostained using an anti-FTO antibody, and the results verified the complete knockout of endothelial FTO protein (Figure 2B). In addition, the genomic DNA of primary mouse retinal ECs was extracted and analyzed using PCR, and the results confirmed successful cre-mediated recombination of the *Fto* locus in EC *Fto*^Δ/Δ^ mice (band size, ~757 bp; Figure 2C). Further Western blotting validated the depletion of FTO protein in the ECs from EC *Fto*^Δ/Δ^ mice (Figure 2D). In addition, dot blot assay indicated increased levels of m^6^A in EC *Fto*^Δ/Δ^ mice (Figure 2E). We explored the functional role of FTO in diabetes-induced retinal vascular endothelial dysfunction. Evans blue leakage assays revealed that endothelial depletion of *Fto* alleviated diabetes-induced retinal vascular leakage (Figure 2F). In line with this, EC *Fto*^Δ/Δ^ mice displayed fewer acellular capillaries in retinas (Figure 2G). In addition, lower levels of inflammatory factors, including IL-1β and IL-18, were detected by ELISAs in the retinas of EC *Fto*^Δ/Δ^ mice after introduction of diabetes (Supplemental Figure 2A).

In vitro, HRMECs were transfected with overexpression plasmid or siRNA of *FTO*. First, qRT-PCR was conducted to examine overexpression (Supplemental Figure 2B) or knockdown (Supplemental Figure 2C) efficiency, which were confirmed by Western blotting assays (Supplemental Figure 2, D and E). High glucose could enhance inflammation cytokine (IL-1β, IL-18) secretion (Supplemental Figure 2F), migration ability (Figure 2H), tube formation (Figure 2I), proliferation (Supplemental Figure 2G), and apoptosis (Supplemental Figure 2H) of HRMECs; however, this trend could be reversed by silencing *FTO*. To the contrary, sustained *FTO* increased apoptosis (Supplemental Figure 2I) and aggravated inflammation (Supplemental Figure 2J) in HRMECs.

The consistent trend of elevated FTO stressed by high glucose was also validated in both mouse cardiac ECs (MECEs) (Supplemental Figure 3A) and mouse renal glomerular ECs (MRGECs) (Supplemental Figure 3B). Furthermore, silencing FTO inhibited inflammation cytokines and tube formation induced by high glucose in both MECEs (Supplemental Figure 3, C and E) and MRGECs (Supplemental Figure 3, D and F). Taken together, FTO aggravates diabetes-related retinal vascular dysfunction and inflammation both in vivo and in vitro.

### Tnip1 is a potential FTO-mediated target identified by MeRIP-Seq combined with RNA-Seq.

**Figure 3:**
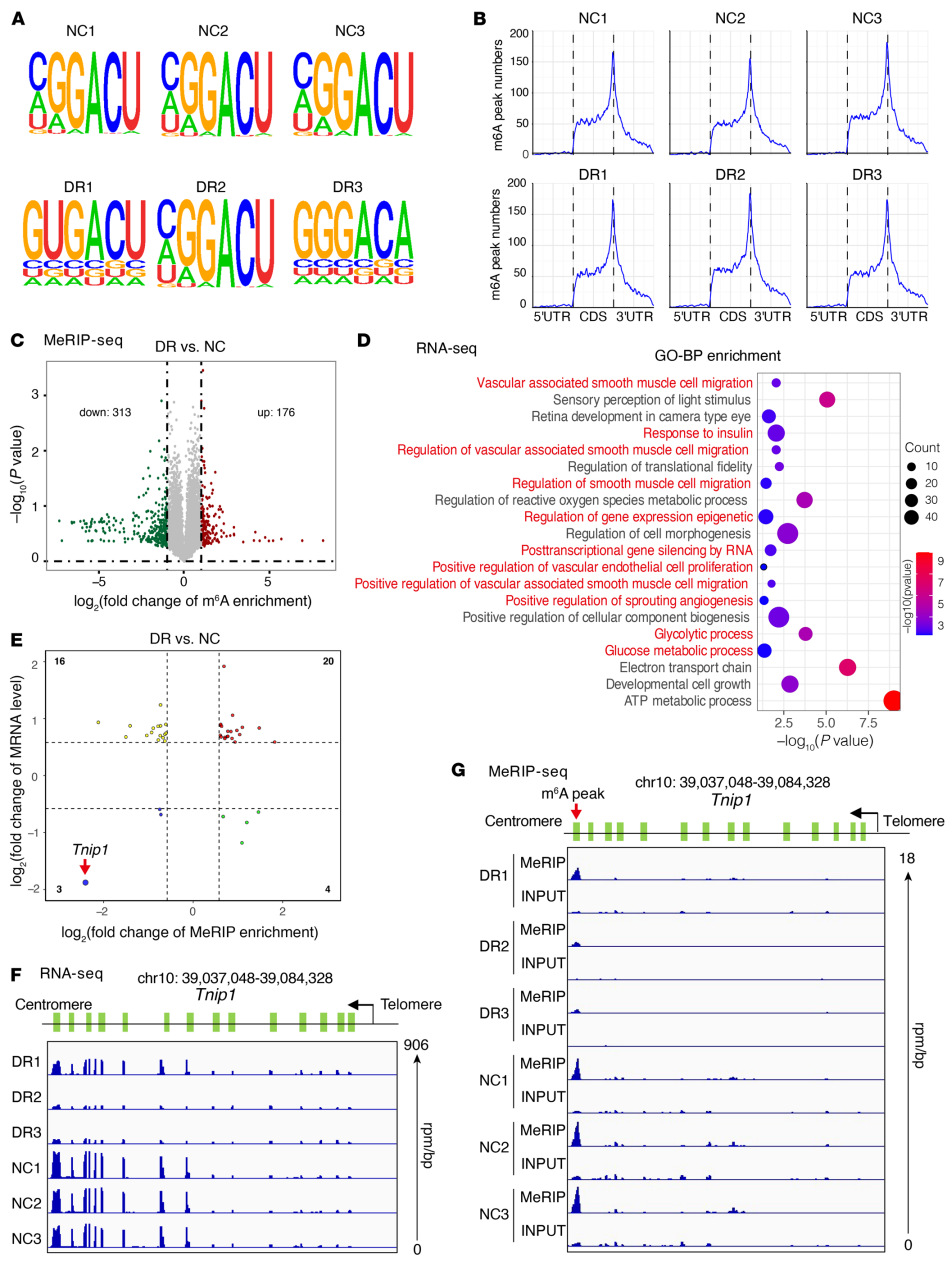
Tnip1 is the target of m^6^A revealed by transcriptome-wide identification. (**A**) Top enriched motifs of m^6^A peaks identified in diabetic and normal retinas. Samples from normal controls are numbered 1–3, as are samples from murine retinas with diabetic retinopathy. CDS, coding sequences. (**B**) Distribution of m^6^A sites plotted by mRNA transcripts. (**C**) Volcano plot showing m^6^A enrichment of genes in diabetic retinas. (**D**) Gene ontology (GO) analysis based on RNA-Seq for differentially expressed genes in diabetic retinas. The pathways in red are highly related to “Glucose metabolic process,” “Angiogenesis,” and “Epigenetic regulation.” (**E**) A plot indicating the m^6^A enrichment and mRNA expression of differentially expressed genes in diabetic retinas. *Tnip1* is denoted for its remarkable demethylation and reduced level of mRNA. (**F**) Gene tracks based on RNA-Seq of *Tnip1* using Integrative Genomics Viewer (IGV) in normal and diabetic murine retinas. rpm/bp, reads per million mapped reads per base pair. (**G**) Gene tracks based on MeRIP-Seq of *Tnip1* using IGV in normal and diabetic murine retinas. DR, diabetic retinopathy.

Methylated RNA immunoprecipitation sequencing (MeRIP-Seq) combined with RNA-Seq, with 3 independent biological replicates, was performed to predict the target of m^6^A modification in diabetes (GEO GSE176355). In MeRIP-Seq, m^6^A peaks were detected in the canonical RRACH motif (Figure 3A) and were enriched in the 3′-untranslated region (UTR) approaching the stop codons (Figure 3B and Supplemental Figure 4A). The distribution of genes with different numbers of m^6^A sites is displayed in Supplemental Figure 4B. Compared with normal retinas, under diabetic conditions, 313 transcripts had fewer enriched m^6^A modifications, and 176 transcripts had increased m^6^A peak enrichments (Figure 3C). Gene ontology (GO) analysis indicated that differentially expressed genes were mostly enriched in several signaling pathways, including the response to insulin, positive regulation of vascular EC proliferation, and glucose metabolic process, etc. (Figure 3D). Further GO (Supplemental Figure 4C) and Kyoto Encyclopedia of Genes and Genomes (KEGG; https://www.genome.jp/legg/) (Supplemental Figure 4D) analyses were conducted for differential genes in both RNA-Seq and MeRIP-Seq.

Combined RNA-Seq and MeRIP-Seq analyses predicted 43 differentially expressed genes in diabetes (Supplemental Table 1), and 12 of them may serve as a direct effector of FTO (Supplemental Table 2). *Tnip1*, with most remarkably reduced levels of mRNA and m^6^A peaks, was identified as the target of m^6^A modifications stressed by diabetes (Figure 3E). Compared with normal retinas, diabetic samples displayed fewer reads (Figure 3F and Supplemental Figure 4E) and m^6^A peaks in the 3′-UTR of *Tnip1* mRNA (Figure 3G). Consequently, we speculated that FTO causes retinal vascular endothelial dysfunction in diabetes possibly through the RNA demethylation of *Tnip1*.

### TNIP1 alleviates diabetes-induced retinal vascular endothelial dysfunction.

**Figure 4:**
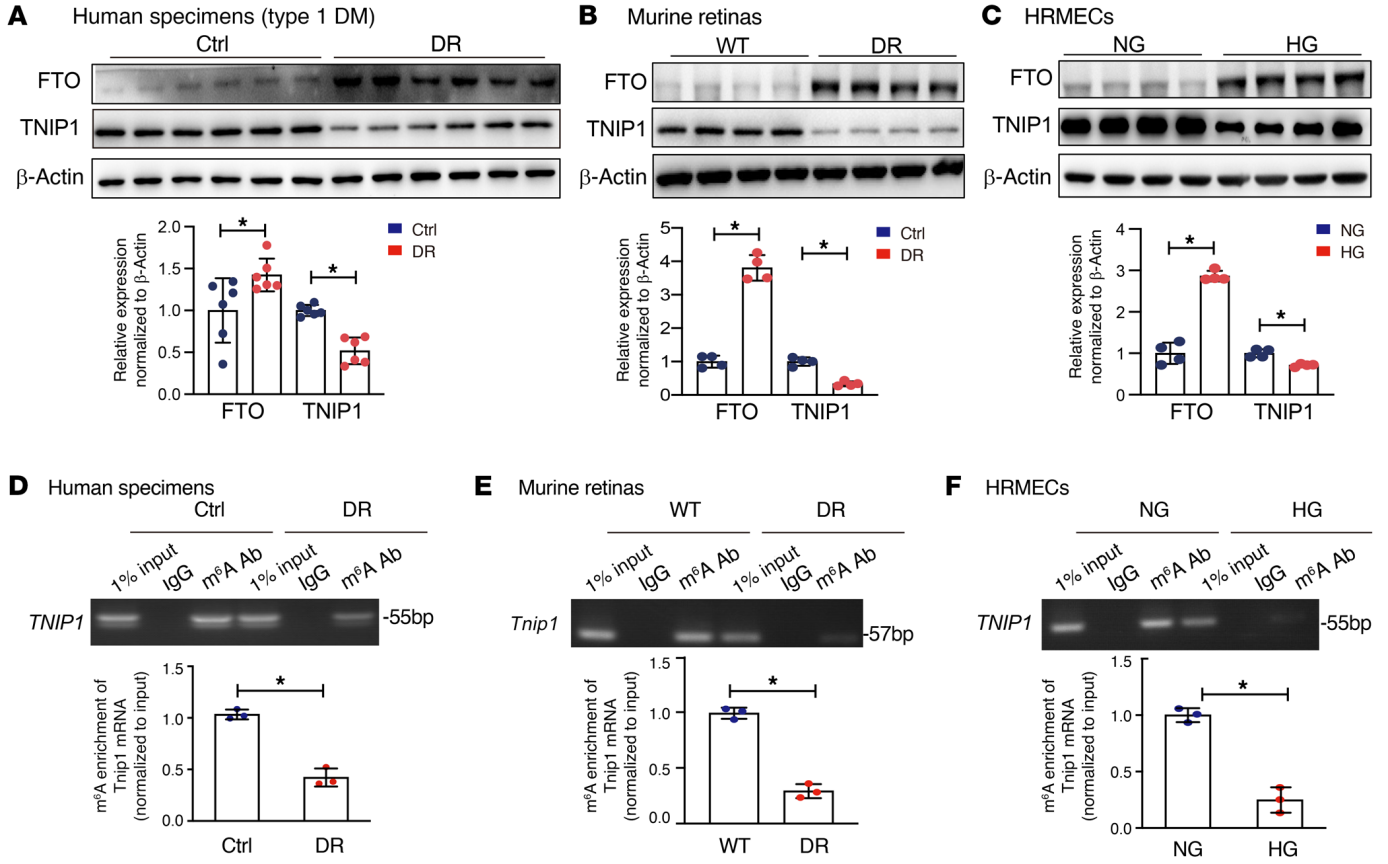
The level of *TNIP1* and its m^6^A modification are reduced in diabetic condition. (**A**–**C**) Reduced TNIP1 and enhanced FTO were detected by Western blotting in the retinal fibrovascular membranes of patients with retinopathy due to type 1 diabetes (**A**, *n* = 6), diabetic mouse retinas (**B**, *n* = 4), and human retinal microvascular endothelial cells (HRMECs) cultured in high glucose (**C**, *n* = 4) (Student’s *t* test). (**D**–**F**) Reduced m^6^A modification of *TNIP1* transcripts in the retinal fibrovascular membranes of patients with diabetic retinopathy (**D**, *n* = 3), diabetic mouse retinas (**E**, *n* = 3), and HRMECs treated with high glucose (**F**, *n* = 3), as assessed by m^6^A-RIP-qPCR assays. The value obtained for control group was set to 1 (Student’s *t* test). NG, normal glucose (5.5 mM) with D-mannitol as osmotic control; HG, high glucose (30 mM). *P < 0.05.

To confirm the sequencing findings, we proved that the protein level of TNIP1 was decreased, accompanied by upregulated FTO, in the retinal fibrovascular membranes of the patients with diabetic retinopathy (Figure 4A and Supplemental Figure 5A), diabetic murine retinas (Figure 4B), and HRMECs treated with high glucose (Figure 4C). Suppressed mRNA levels of *Tnip1* were also detected in diabetic murine retinas (Supplemental Figure 5B) and HRMECs treated with high glucose (Supplemental Figure 5C). Moreover, decreased m^6^A modifications of *TNIP1* mRNA were also identified by RNA immunoprecipitation-qPCR (RIP-qPCR) in human retinal fibrovascular membranes (Figure 4D), murine retinas (Figure 4E) and HRMECs (Figure 4F) under diabetic conditions.

**Figure 5:**
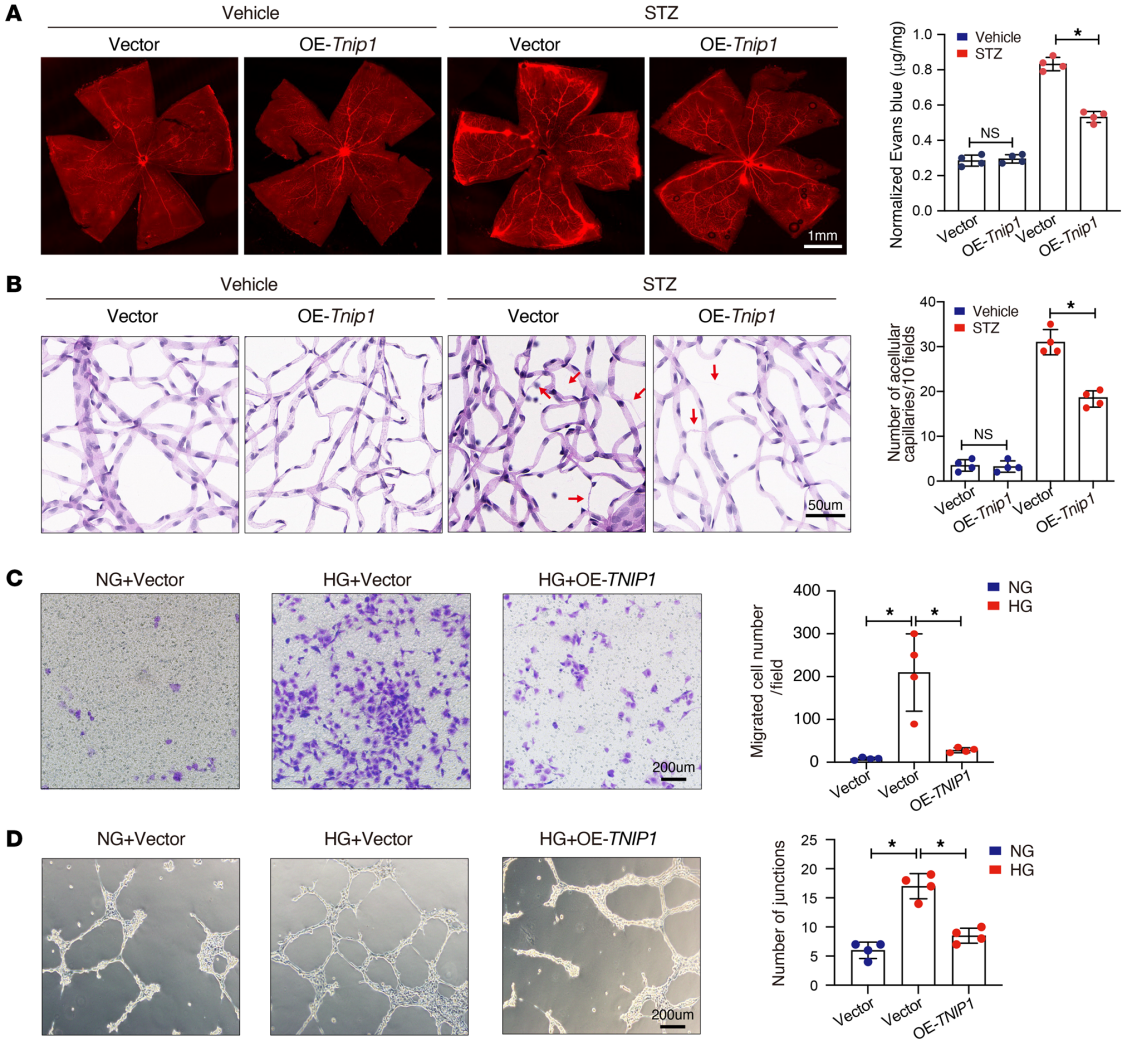
Tnip1 alleviates retinal vascular endothelial dysfunction in diabetic mice. (**A**) *Tnip1* alleviates diabetes-induced retinal endothelium vascular leakage, as observed by staining flat-mounted retinas with Evans blue dye. A representative image with the quantification of the fluorescence signal is shown (*n* = 4, scale bar: 1 mm). (**B**) *Tnip1* attenuated acellular retinal capillary formation in diabetes, as indicated by retinal trypsin digestion. Red arrows indicate acellular capillaries. Acellular capillaries were quantified in 20 high-power fields and averaged (*n* = 4, scale bar: 50 μm). (**C**) Transwell assays showing that *TNIP1* decreased the migration ability of HRMECs treated by high glucose. The number of migrated cells was quantified (*n* = 4, scale bar: 200 μm). (**D**) *TNIP1* inhibited tube formation of HRMECs cultured in high glucose. The average number of tube formation for each field was assessed (*n* = 4, scale bar: 200 μm). NG, normal glucose (5.5 mM) with D-mannitol as osmotic control; HG, high glucose (30 mM). Significant differences were calculated by 1-way ANOVA or Kruskal-Wallis’s test followed by Bonferroni’s post hoc comparison test. Data are shown as the mean ± SD. *P < 0.05.

We further determined the effect of TNIP1 on diabetes-induced retinal vascular dysfunction. The expression of TNIP1 could be modulated by intravitreal injection of AAV vectors overexpressing *Tnip1* (Supplemental Figure 5D) or silencing it (Supplemental Figure 5E) in retinal microvascular ECs, as indicated by immunofluorescence assays (Supplemental Figure 5F). Sustained expression of *Tnip1* in the retinas significantly reduced retinal vascular leakage (Figure 5A) and the number of acellular capillaries (Figure 5B).

In vitro, HRMECs were transfected with overexpression plasmid or siRNA of *TNIP1*. qRT-PCR verified successful overexpression (Supplemental Figure 5G) or knockdown (Supplemental Figure 5H) of *TNIP1*. Moreover, *TNIP1* remarkably inhibited migration (Figure 5C) and tube formation (Figure 5D) in HRMECs cultured in high glucose. In addition, strengthened *TNIP1* significantly ameliorated the enrichment of IL-1β and IL-18 (Supplemental Figure 5I), proliferation (Supplemental Figure 5J), and apoptosis (Supplemental Figure 5K) induced by high glucose. Collectively, these results indicate that *TNIP1* attenuates diabetes-related retinal vascular dysfunction and inflammation both in vivo and in vitro.

### The FTO-TNIP1-NF-κB network regulates diabetes-induced retinal vascular endothelial dysfunction.

**Figure 6:**
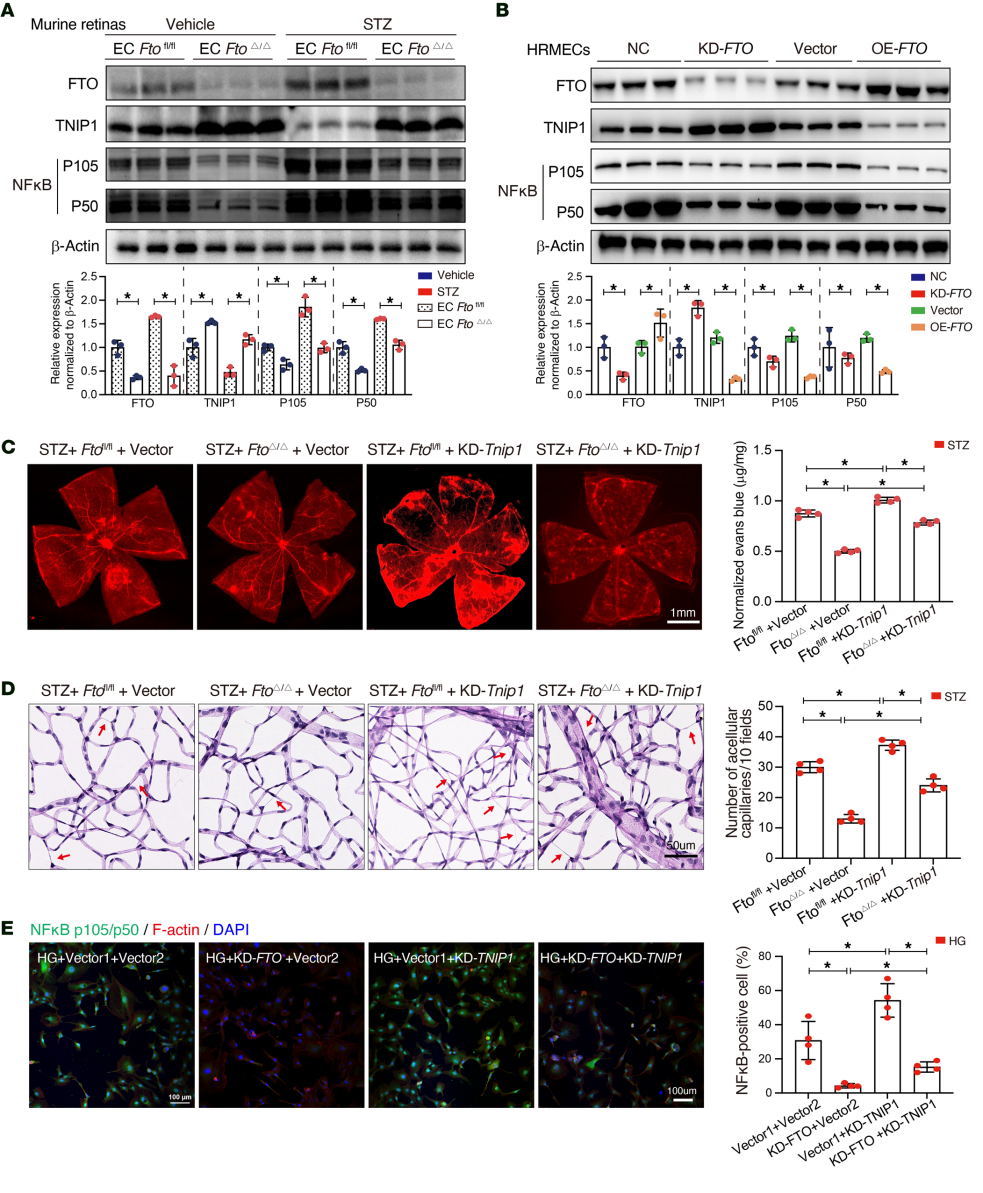
The FTO-TNIP1-NF-κB network regulates diabetes-induced retinal vascular endothelial dysfunction. (**A**) Western blotting displaying higher TNIP1 expression and lower NF-κB expression in the retinas of endothelial cell–specific (EC-specific) *Fto*-deficient (EC *Fto*^Δ/Δ^) mice as compared with EC *Ft*o^fl/fl^ mice (*n* = 3). (**B**) Western blotting indicating that TNIP1 was inversely correlated with FTO expression, while the expression of NF-κB positively changed with FTO (*n* = 3). (**C**) Silencing *Tnip1* by the intravitreal injection of adeno-associated virus (AAV) vectors containing siRNA-*Tnip1* increased retinal vascular leakage in EC *Fto*^Δ/Δ^ mice (*n* = 4, scale bar: 1 mm). (**D**) Silencing *Tnip1* by the intravitreal injection of AAV vectors containing siRNA-*Tnip1* increased the number of acellular capillaries in EC *Fto*^Δ/Δ^ mice (*n* = 4, scale bar: 50 μm). (**E**) Immunofluorescence showing that downregulated FTO suppressed NF-κB, and this trend was reversed by silencing *TNIP1* (*n* = 4, scale bar: 100 μm). Significant differences were assessed by 1-way ANOVA followed by Bonferroni’s post hoc comparison test. Data are shown as the mean ± SD. *P < 0.05.

TNIP1, a suppressor of the NF-κB pathway, is involved in antiinflammatory response and autoimmunity. We hypothesized that FTO regulates the TNIP1-NF-κB pathway, activated inflammatory cytokines, and, finally, caused endothelial damage. To validate this mechanism, first, the protein levels of FTO, TNIP1, and NF-κB were examined in the retinas of both EC *Fto*^fl/fl^ and EC *Fto*^Δ/Δ^ mice. The results revealed upregulated FTO, accompanied by decreased TNIP1 and elevated NF-κB (p105/p50) levels in EC *Fto*^fl/fl^ mice with diabetes, whereas in EC *Fto*^Δ/Δ^ mice, upregulated TNIP1 with suppressed NF-κB was observed (Figure 6A). By modulating *FTO* expression through transfecting siRNA or overexpression plasmid in HRMECs, the protein (Figure 6B) and mRNA levels (Supplemental Figure 6A) of TNIP1 were inversely correlated with FTO expression, while NF-κB positively changed with FTO. Silencing *Tnip1* by intravitreal injection of AAV vectors exacerbated retinal vascular leakage (Figure 6C) and acellular capillaries formation (Figure 6D) in EC *Fto*^Δ/Δ^ mice. Consistently, the knockdown of *TNIP1* increased inflammatory levels of IL-1β and IL-18 (Supplemental Figure 6B), tube formation (Supplemental Figure 6C), and apoptosis rate (Supplemental Figure 6D) after inhibiting *FTO* in HRMECs. Moreover, immunofluorescence analysis showed that inhibited *FTO* downregulated NF-κB expression in high glucose, and this effect became unremarkable after *TNIP1* knockdown (Figure 6E).

It is known that deubiquitination of NF-κB essential modulator (NEMO, also named IKKγ) blocks NF-κB gene regulation. A20-mediated removal of ubiquitin from IKKγ is facilitated by Tnip1. To delineate the regulation of the NF-κB pathway by TNIP1 in diabetes, as TNIP1 is an A20 binding protein, we first used a coimmunoprecipitation assay in HRMECs to detect the direct interaction among TNIP1, A20 (Supplemental Figure 6E), and IKKγ (Supplemental Figure 6F). Furthermore, we proved that Tnip1 promoted A20-mediated deubiquitination of IKKγ (Supplemental Figure 6G), thus resulting in NF-κB suppression.

### FTO regulates TNIP1 RNA expression by m^6^A modification.

**Figure 7:**
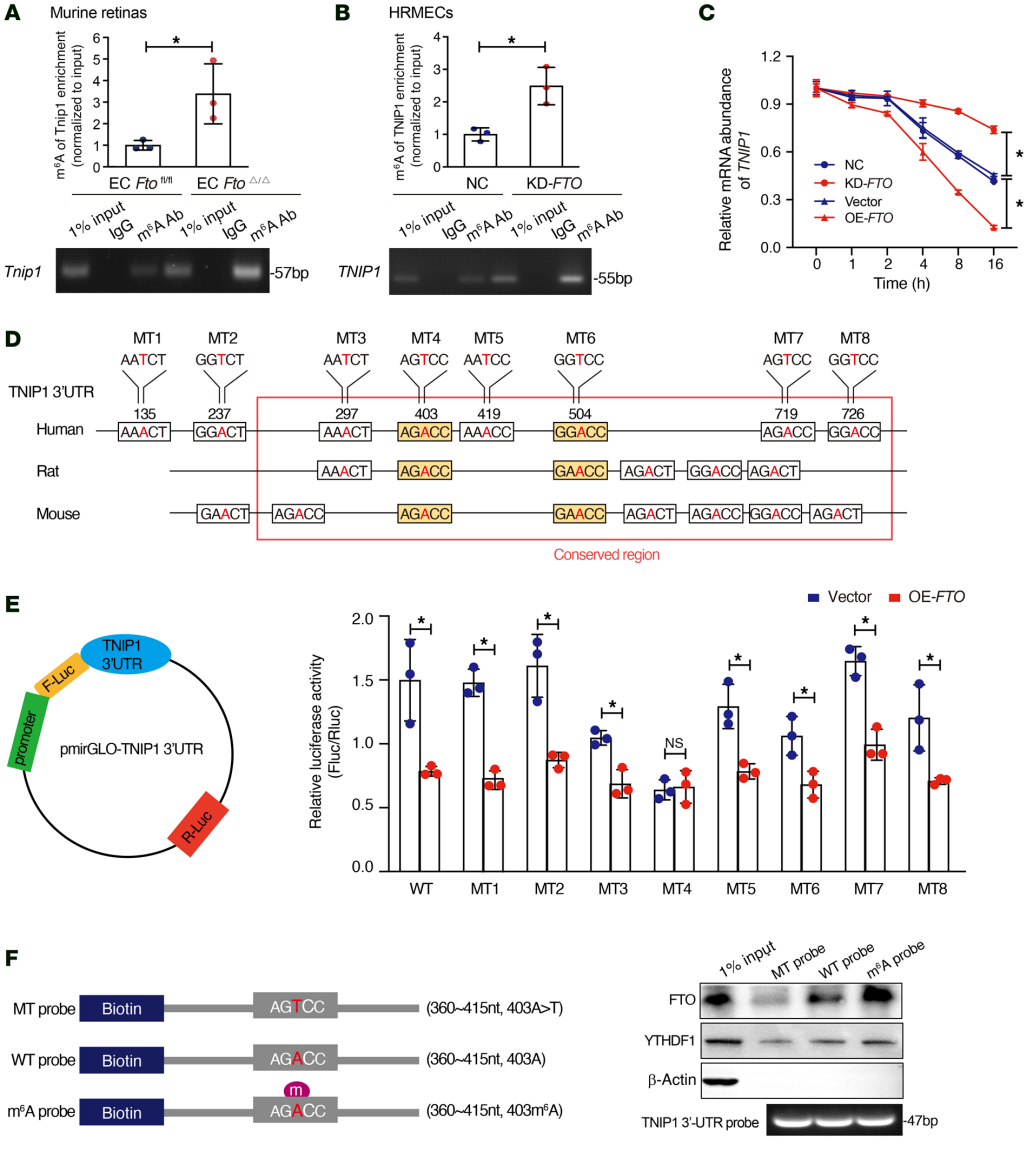
FTO regulates Tnip1 expression by m^6^A modification. (**A**) m^6^A-RIP-qPCR assays showed enhanced m^6^A modification in *Tnip1* transcripts in endothelial cell (EC) *Fto*^Δ/Δ^ mice as compared with EC) *Fto*^fl/fl^ mice. The value obtained for the control group was set to 1 (*n* = 3, Mann-Whitney *U* test). (**B**) Elevated m^6^A modification in *TNIP1*1 transcript after *FTO* knockdown, as assessed by gene-specific m^6^A-RIP-qPCR assays, in human retinal microvascular ECs (HRMECs). The value obtained for the control group was set to 1 (*n* = 3, Mann-Whitney *U* test). (**C**) qRT-PCR was conducted to detect *TNIP1* mRNA after actinomycin D treatment (*n* = 3, repeated-measures ANOVA followed by Bonferroni’s test). (**D**) Schematic diagram depicting 8 mutants used in luciferase reporter assays, which are located in the *TNIP1* 3′ UTR of human and murine genomes. (**E**) Dual luciferase reporter assays showed the effect of overexpressed *FTO* on *TNIP1* mRNA reporters with either wild-type or mutated m^6^A sites (*n* = 3, Mann-Whitney *U* test). (**F**) Left: A schematic model showing RNA probes used in RNA pull-down assays. Right: RNA pulldown of endogenous FTO proteins using synthetic *TNIP1* RNA fragments with or without m^6^A modifications. FTO selectively recognized the dynamic m^6^A modification to regulate the lifetime of *TNIP1* mRNA with the positive reference of YTHDF1.

To investigate the mechanisms underlying FTO-mediated m^6^A modification of TNIP1, RIP assays were performed, and the results revealed that compared with EC *Fto*^fl/fl^ mice, EC *Fto*^Δ/Δ^ mice had enriched m^6^A modification of *Tnip1* mRNA (Figure 7A). Consistently, the m^6^A level of *TNIP1* mRNA was significantly higher after silencing FTO in HRMECs (Figure 7B). Furthermore, the stability of *TNIP1* mRNA was examined after the administration of actinomycin D, an inhibitor of transcription. The qRT-PCR results revealed that the half-life of *TNIP1* was approximately 10 hours, and this time was extended by silencing *FTO* while it was shortened with enhanced *FTO* (Figure 7C). Reporters constructed with the *TNIP1* 3′-UTR or the 8 mutated (MT) sequences of the m^6^A locus were created, and MT3–MT8 were located in a highly conserved region among the human, murine, and rat genomes (Figure 7D). Gene homology comparisons of *TNIP1* 3′-UTR between human and mouse or rat are showed in Supplemental Figures 7 and 8, respectively. Among the 8 missense mutations of *TNIP1* that were investigated, MT4 (403A>403T) lost the ability to inhibit the transcription of *TNIP1*, and the other 7 mutants remained unchanged from the wild-type (Figure 7E). Moreover, RNA pull-down assays confirmed that FTO selectively recognized the dynamic m^6^A modification to regulate the lifetime of *TNIP1* mRNA with YTHDF1 as a positive reference (Figure 7F).

## Discussion

**Figure 8:**
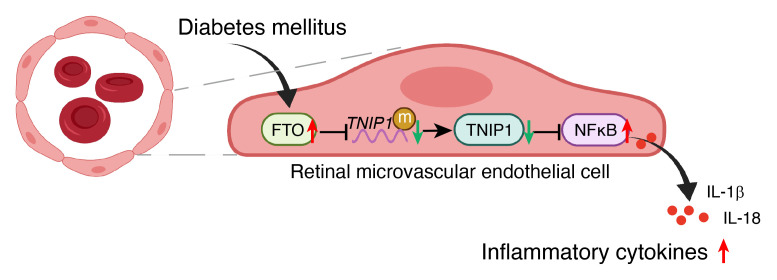
Schematic diagram illustrating the mechanisms underlying the regulation of the FTO-TNIP1-NF-κB network in diabetes-induced retinal vascular endothelial dysfunction associated with inflammation. In diabetes, excessive FTO expression leads to m^6^A demethylation of *TNIP1* mRNA. TNIP1 depletion activates the NF-κB pathway and subsequently elevates the inflammatory cytokines, such as IL-1β and IL-18, finally leading to vascular endothelial dysfunction.

This study investigated m^6^A modification underlying diabetic vascular complications and initially demonstrated that dysregulated FTO-guided m^6^A hypomodifications are an important trigger of inflammation in diabetes-induced vascular endothelial dysfunction. In diabetes, excessive FTO downregulates *TNIP1*, a central regulator of inflammation, thereby activating NF-κB and subsequently increasing inflammatory cytokines (IL-1β and IL-18) in an m^6^A-dependent manner, ultimately leading to vascular endothelial dysfunction (Figure 8).

Retinal vessel anomalies, which can be observed and evaluated noninvasively by direct ophthalmoscopy, are generally the early signs of endothelial damage induced by hyperglycemia, suggesting the onset of retinopathy and other diabetes-related vascular complications. Given that endothelial dysfunction is the common initiating factor of different diabetic complications, early detection and intervention of endothelial abnormalities can prevent not only diabetic retinopathy, but also other vascular complications caused by diabetes (24). Although the mechanisms by which diabetes contributes to endothelial dysfunction are unclear, it is likely that chronic low-grade inflammation may plays a vital role throughout this process (4, 25–28).

In this study, we found that diabetes-induced vascular endothelial dysfunction was associated with increased levels of inflammatory cytokines, such as IL-1β and IL-18, which were mediated by the FTO-TNIP1-NF-κB network. Essentially, NF-κB is known to trigger vascular inflammation, insulin resistance, and glucose homeostasis in diabetes (29–31). Moreover, by comparing the diabetic m^6^A maps of individual transcripts, it was discovered that FTO selectively demethylates inflammatory transcripts, such as *Galnt3*, *Arhgap15*, *Vamp3*, and *Trim8*, decreasing their mRNA stability. *Tnip1* had the most significant reduction in both m^6^A peaks and mRNA levels in diabetes. TNIP1, also referred to as A20 binding and inhibitor of NF-κB (ABIN-1), is a negative regulator of NF-κB signaling (32, 33). In the classic NF-κB pathway, TNF-αR recruits intracellular proteins to form a complex, which facilitates the phosphorylation and subsequent polyubiquitination of IKKγ. Then, IκB is targeted for degradation, allowing the heterodimer of p65/p50 NF-κB to translocate into the nucleus, and NF-κB signaling is activated thereafter. TNIP1 specifically binds IKKγ to facilitate A20-mediated deubiquitination of IKKγ, which sustains IκB, and finally prevents the nuclear translocation of p65/p50 (32, 33). In addition, TNIP1 inhibits the processing of p105 to the subunit p50 and, therefore, blocks the activation of NF-κB. Moreover, TNIP1 prevents programmed cell death by inhibiting caspase-8 recruitment to FADD in TNF-induced signaling complexes (34).

Clinically, TNIP1 is known to play critical roles in the antiinflammatory response and tissue homeostasis (34). Previous studies have indicated that mutations in *TNIP1* are associated with psoriasis (35), rheumatoid arthritis (36), systemic lupus erythematosus (37), and leukemia and lymphoma (38, 39). Notably, *Tnip1* sustains cell survival and embryonic development, and *Tnip1* deficiency causes embryonic lethality with fetal liver apoptosis, anemia, and hypoplasia (34).

In this study, sustained expression of *Tnip1* using an AAV vector attenuated diabetes-induced vascular dysfunction in vivo, demonstrating its therapeutic potential for the management of diabetes-associated vascular complications. Our findings indicated that, in diabetes, TNIP1 physically links A20 to IKKγ and facilitates A20-mediated deubiquitination of IKKγ, thus resulting in the suppression of NF-κB. This process can be modulated by FTO through m^6^A demethylation. FTO confers selective demethylation in high glucose conditions, which decreases both the mRNA stability and protein expression level of TNIP1. RNA pulldown assay proved the direct binding of FTO and *Tnip1* mRNA, and dual luciferase assays identified the binding site of adenine that was demethylated by FTO. This finding was also confirmed by RNA pulldown. Collectively, our data show that FTO-TNIP1-NF-κB, a previously unidentified pathway to our knowledge, mediates diabetes-induced vascular endothelial changes via the RNA hypomethylation.

In the present study, TNIP1-NF-κB–mediated endothelial inflammation in diabetes was regulated by FTO. FTO has been found to play a pivotal role in regulating transcriptome-wide m^6^A modification of mRNA, and it is one of demethylases that has been associated with metabolic disorders, such as diabetes, obesity, and cardiovascular disease (19, 40, 41). Previous studies have shown reduced m^6^A methylation in diabetes, and this was attributed to overexpressed FTO rather than ALKBH5 (20). FTO is not only involved in obesity-related diseases, but it also plays a critical role in neurodegenerative disorders (42) and various cancers, such as lung cancer (43), gastric cancer (44), acute myeloid leukemia (45), and melanoma (46).

Our murine studies proved a causative role of FTO in retinal ECs, as shown by EC *Fto*^Δ/Δ^ mice, which were protected against severe diabetic retinopathy, and we additionally linked FTO to inflammation alterations. By regulating *FTO* expression through silencing or overexpressing it, we have also validated that FTO is a key contributor of global m^6^A levels in high glucose condition and it directly aggravates the vascular leakage, acellular capillary formation, and inflammation.

It is well known that inactivation of the *FTO* gene protects against obesity (21). A recent study using endothelial *Fto*-deficient mice proved that loss of endothelial *Fto* did not induce obesity and dyslipidemia, as it protected mice from high-fat diet–induced glucose intolerance and insulin resistance (22). Another study showed that FTO promoted endothelial angiogenesis in murine hearts after myocardial infarction, which was beneficial for myocardial repair and remodeling (47). Notably, FTO plays a context-dependent role under different conditions. In this research, we observed elevated FTO stressed by high glucose not only in the ECs of retina, but also in other target organs of diabetes, such as heart and kidney. Furthermore, we described that endothelial loss of FTO alleviated diabetes-induced inflammation and angiogenesis in multiple ECs. Presumably, the mechanisms, by which, FTO controls vascular changes in retinopathy also underlies other micro- as well as macrovascular complications of diabetes. These data highlight that FTO mediates essential vascular effects and, for the first time to our knowledge, connect FTO to key regulators of inflammation in vascular complications of diabetes.

This study should be regarded as an initial exploration of the mechanisms of FTO-dependent RNA demethylation in diabetes-induced endothelial dysfunction. However, several limitations hinder the interpretation of our findings. First, the RNA-Seq results indicated that, in addition to *Fto* overexpression in diabetes, *Mettl3* was slightly downregulated, suggesting that this writer may also contribute to reduced m^6^A in diabetes. m^6^A is dynamically modulated by writers, erasers, and readers. The interweaving effect among these proteins is also critical for regulating diabetes-associated endothelial changes. Whether METTL3 cooperates or competes with FTO or functions in a mutually exclusive manner has yet to be elucidated. In addition, MeRIP-Seq was utilized to map transcriptome-wide m^6^A, and we discovered several other inflammation pathways other than *Tnip1*. Moreover, rescue experiments suggested that other downstream genes regulated by FTO in endothelial dysfunction exist; however, the pathway that plays the dominant role remains unclear.

In summary, we have found in vitro, in vivo, and translational evidence demonstrating a pivotal role for the FTO-TNIP1-NF-κB pathway in regulating endothelial dysfunction caused by diabetes. FTO-mediated RNA demethylation of *TNIP1* activates NF-κB, thus accelerating diabetes-induced vascular endothelial dysfunction. Our data provide important translational implications for targeting the key elements of this pathway to prevent endothelial damage in diabetes. However, further studies and clinical trials are warranted to fully validate the mechanisms underlying the FTO-mediated benefits in the setting of diabetes and subsequent vascular complications.

## Methods

### Patient samples.

Thirty patients with proliferative diabetic retinopathy (PDR) due to type 1 (*n* = 10) or type 2 (*n* = 20) diabetes were recruited at Shanghai General Hospital from May 2022 to January 2023. Another 30 patients with idiopathic epiretinal membrane were selected to serve as controls. A characteristic pathological sign of PDR is the formation of fibrovascular membranes on the surface of retina. Retinal fibrovascular membranes are formed by the migration and proliferation of vascular ECs (48, 49), which causes vitreous hemorrhage and tractional retinal detachment and, ultimately, leads to blindness. In contrast, idiopathic epiretinal membrane is characterized by a fibroproliferative membrane, which occurs in the vitreoretinal junction; this is also a common disturbance of central vision (50). The indications for vitrectomy for PDR are nonclearing vitreous hemorrhage and/or tractional retinal detachment involving the foveal. During the vitrectomy, retinal fibrovascular membranes were collected from patients with diabetic retinopathy, and epiretinal membranes were obtained from people in the control group. The surgical specimens were handled and stored at –80°C for experiments. A diagram showing patient sample collection is shown in Supplemental Figure 1A. The demographic and baseline characteristics of patients are detailed in Supplemental Table 3.

### Generation of EC Fto^Δ/Δ^ mice.

The comparison of *FTO* genes among humans, mice, and rats implied that *FTO* is highly conserved across species (Supplemental Figures 9–11). Accumulating evidence has showed the conservation of *FTO* function controlling vascular changes in humans and other species (22, 47). Therefore, EC *Fto*^Δ/Δ^ mice were generated to explore the role of *Fto* in endothelial dysfunction. EC *Fto*^Δ/Δ^ mice were constructed using CRISPR/Cas9 technology (51). Brieﬂy, *Fto*-floxed mice, background in C57BL/6, were constructed using guide RNAs and Cas9 mRNA to insert the loxP sites upstream and downstream of *Fto* exon 3 to induce frameshift mutation (21), and heterozygous *Fto*^fl/+^ mice were self-crossed to generate homozygous *Fto*^fl/fl^ mice. Then, the *Fto*^fl/fl^ mice were crossed with *Cdh5*-Cre transgenic mice (The Jackson Laboratory) to generate *Fto*^fl/fl^ *Cdh5*-Cre mice for experiments. After derivation, EC *Fto*^Δ/Δ^ mice were housed in specific pathogen–free conditions at Shanghai Model Organisms Center (Shanghai, China). Following genotyping, backcrossed mice were divided into EC *Fto*^fl/fl^ and EC *Fto*^Δ/Δ^ groups. For verification of successful cre-mediated recombination of the *Fto* locus, the primer for EC *Fto*^Δ/Δ^ was used (band size: ~757 bp). The primers are listed in Supplemental Table 5.

### Diabetic mice.

To induce the model of type 1 diabetes, for C57BL/6 mice (8 week old, male), either STZ (Sigma-Aldrich; 60 mg/kg STZ after fasting for 6 hours) or vehicle (citrate buffer control) was injected intraperitoneally for 5 consecutive days. Fasting blood glucose was tested for 1 week after the last STZ injection, and values of more than 16.7 mmol/L were considered diabetic.

To induce the model of type 2 diabetes, mice were fed with high-fat diet while being treated with STZ. Briefly, the 8-week-old C57BL/6 male mice were fed with high-fat diet for 4 weeks and then intraperitoneally injected STZ (Sigma-Aldrich, 50 mg/kg STZ after fasting for 6 hours). The STZ injection was conducted for 5 days while the mice drank 10% glucose solution during the treatment. The control mice were injected citrate buffer and fed normal chow. All mice were maintained on their respective diets till sacrifice. Fasting blood glucose was tested 1 week after the last STZ injection, and values of more than 16.7 mmol/L were considered diabetic. Retinal vascular phenotype experiments, including Evans blue dye leakage and trypsin digest assays, were performed 3 months after diabetes induction (Supplemental Figure 1B).

### Intravitreal injection.

AAV serotype 2 (AAV2) (Hanheng Biotechnology), specific to retinal tissue, containing *Fto* siRNA, OE *Fto*, *Tnip1* siRNA, OE *Tnip1*, or scrambled guide RNA was injected intravitreally under general anesthesia, induced by intraperitoneal injection of ketamine (80 mg/kg) and xylazine (4 mg/kg). Recombinant AAV harbored a green fluorescence protein and a promoter of *Tie2*, which is specific to ECs. AAV was plaque purified, and the original titer was 1 × 10^10^ vg/μL. First, the total volume delivered was 1 μL, containing different concentrations (1 × 10^8^ vg/μL, 1 × 10^9^ vg/μL, 1 × 10^10^ vg/μL) of the AAV tested. The AAV with the concentration of 1 × 10^9^ vg/μL was used in the following experiments. One hour before general anesthesia, eyes were dilated with 0.5% tropicamide eye drops, followed by topical administration of 2.5% phenylephrine (Santen Pharmaceutical Co. Ltd.). AAV vector (1 × 10^9^ vg/μL) was injected into the vitreous of mice through a 33-gauge blunt needle in a Hamilton syringe. Intravitreal positioning of the needle was confirmed under a surgical microscope to avoid touching lens. The conversion of recombinant AAV-DNA to a transcriptionally active double-stranded form needs nearly 2 weeks; therefore, AAV vectors were injected intravitreally 2 weeks before diabetes induction. The oligonucleotide sequences are provided in Supplemental Table 4.

### Retinal trypsin digestion assay and staining.

The eyeballs were enucleated and fixed in 4% paraformaldehyde for 24 hours. The retinas were washed with distilled water, digested using 3% trypsin at 37°C for 3 hours, gently shaken to free the vessel network, washed, and mounted on glass slides for drying. The retinal vasculature was then stained with periodic acid–Schiff and hematoxylin.

### Evans blue dye leak.

Diabetic mice and age-matched nondiabetic littermates were anesthetized with ketamine (80 mg/kg) and xylazine (4 mg/kg). The right jugular vein and right iliac artery were cannulated and then infused with heparinized saline. Evans blue (100 mg/mL, Sigma-Aldrich, E2129) was injected through the jugular vein over 10 seconds. Immediately after injection, the mice became visibly blue, confirming the distribution of the dye. To ensure the complete circulation of the dye, the mice were kept on a warm pad for 2 hours. Then, approximately 100 μL blood was obtained from the anesthetized mice. The animals were infused via the left ventricle with PBS followed by an infusion of 1% paraformaldehyde. Both eyes were enucleated and bisected at the equator. Retinas were dissected carefully and fixed in 4% paraformaldehyde in PBS for 30 minutes at room temperature. The retinas were treated with dimethylformamide overnight at 78°C and then centrifuged at 12,000*g* for 15 minutes. The blood samples were centrifuged at 16,000*g* for 15 minutes at 4°C and diluted 1:100 in formamide prior to spectrometric evaluation. To assess the concentration of Evans blue, the absorbance of the retinal extract and plasma samples was measured at 620 nm (blue) and 740 nm (background). The concentration of dye in the plasma was calculated from a standard curve of Evans blue in formamide.

### RNA m^6^A dot blot assays.

The extracted RNAs were transferred onto a nitrocellulose membrane (Amersham, GE Healthcare) with a Bio-Dot apparatus (Bio-Rad). The membranes were incubated with m^6^A antibody overnight at 4°C and subsequently incubated with HRP-conjugated goat anti-mouse IgG. Finally, the membrane was exposed to Hyperfilm electrochemiluminescence, and images were recorded. Membranes stained with methylene blue were used to ensure consistency among different groups.

### Cell culture and transfection.

HRMECs (catalog ACBRI 181) were purchased from Cell Systems Corporations. MECEs and MRGECs were purchased from Beina Chuanglian Biotechnology Institute (Beijing, China). These cells were cultured in EC medium supplemented with 20% FBS (Gibco), 1% penicillin–streptomycin, 3 ng/mL FGF basic, 10 units/mL heparin with 5% CO_2_ and 95% air at 37°C in a humidified incubator. Primary retinal microvascular ECs were dissected from EC *Fto*^Δ/Δ^ and EC *Fto*^fl/fl^ mice and cultured according to the instructions previously described (52). High glucose was introduced by adding D-glucose to the medium to achieve a final concentration of 30 mM, whereas D-mannitol was used in normal glucose (5.5 mM) to adjust osmotic pressure.

siRNAs targeting *FTO* and the overexpression plasmid for *FTO* (pcDNA3.1-h*FTO*) were purchased from Hanbio Biotechnology. Cells were transfected using Lipofectamine 3000 (Thermo Fisher Scientific) following the manufacturer’s instructions. The oligonucleotide sequences are listed in Supplemental Table 4.

### RNA isolation and qRT-PCR.

Total RNA was extracted from the cells and retinas using TRIzol (Invitrogen) and reverse transcribed using the cDNA PrimeScript RT Reagent Kit (Takara). qRT-PCR was performed using Applied Biosystems Powerup SYBR Green PCR Master Mix (Life Technologies) according to the manufacturer’s protocol, and primers are listed in Supplemental Table 5. All data were normalized to *β-actin*. The 10 μL samples contained 5 μL Power SYBR Green PCR Master Mix (Applied Biosystems), 5 pmol of each primer, 3 μL diethyl pyrocarbonate-treated water, and 1 μL DNA. All assays were run in triplicate. The cycling parameters consisted of 95°C for 10 minutes, followed by 40 cycles of 95°C for 15 seconds, 60°C for 1 minute, and 72°C for 30 seconds. The relative levels of target mRNA expression were calculated using the 2^–ΔΔ^Ct method.

### Western blot and coimmunoprecipitation analysis.

HRMECs or tissues were lysed in buffer consisting of 20 mM HEPES and 0.5% NP-40, 1 mM EDTA, 10 mM pyrophosphate, KOH (pH = 7.5), 150 mM NaCl, 10% glycerol, and protease inhibitors (Roche). The lysates were assayed by SDS-PAGE electrophoresis using precast gels (Bio-Rad) and transferred onto nitrocellulose membranes. After blocking with 5% milk for 1 hour at room temperature, the membrane was incubated with antibody in 5% BSA overnight at 4°C. After incubation with a secondary antibody for 1 hour, the membranes were visualized and quantified using the Odyssey Infrared Imaging System (LI-COR).

HRMECs were rinsed twice with ice-cold PBS and lysed with RIPA buffer containing protease inhibitor on ice for 30 minutes. Then, the cell lysates were obtained by centrifugation at 13,000*g* for 10 minutes. For immunoprecipitations, the supernatants were immunoprecipitated with indicated antibodies overnight at 4°C. The protein G–agarose beads were washed 3 times with lysis buffer and then incubated with the supernatants for 2 hours. After incubation, immunoprecipitated proteins were washed with lysis buffer. Samples were denatured after boiling for 10 minutes with particular sample buffer, then resolved by 8%–16% SDS-PAGE, and finally analyzed by immunoblotting. Antibodies used in this study are listed in Supplemental Table 6.

### ELISA.

The levels of IL-1β and IL-18 in the cells or tissues were determined using a colorimetric ELISA kit (Abcam) according to the manufacturer’s instructions.

### Apoptosis assays.

Apoptosis was assayed by the FITC-Annexin V Apoptosis Detection Kit 1 (BD Biosciences) following the manufacturer’s instructions. Cells were initially washed twice with cold PBS, stained with FITC-Annexin V and PI, and then evaluated by flow cytometric analysis (BD LSRFortessa analyzer).

### Immunofluorescence.

Cells adhered to a glass slide were fixed with 4% formaldehyde for 15 minutes and then blocked with 5% normal goat serum in PBS for 60 minutes at room temperature. The cells were then incubated with anti-FTO, anti-TNIP1, and anti-CD31 antibodies overnight and then incubated with a secondary antibody for 1 hour at room temperature in the dark. After rinsing 3 times with PBS, the slides were incubated with DAPI for 3 minutes and placed in glycerol. Fluorescence was visualized with an inverted microscope (IX53, Olympus).

### Tube formation assay.

Matrigel (BD Bioscience) was added to 24-well plates and then incubated at 37°C for 30 minutes for solidification. The treated cells were resuspended in culture medium and then seeded on the Matrigel at a density of 5 × 10^4^/well. The plates were incubated at 37°C. Six hours later, tube formation was evaluated using an inverted microscope. The images of tube formation were observed at 4 time points (6, 12, 24, and 48 hours) and quantified at 6 hours.

### Migration assay.

The migratory ability of the cells was assessed using Transwell filter membrane chambers (pore size, 8 μm; Millipore). Approximately 1 × 10^5^ cells were seeded into the upper compartment filled with medium supplemented with 2% FBS, and the lower compartment contained medium supplemented with 10% FBS. After 24 hours of incubation at 37°C, the cells on the lower surface of the filter membrane were fixed with methanol and stained with 0.1% crystal violet. Migrated cells were counted by ImageJ software (NIH).

### 5-Ethynyl-2′-deoxyuridine assay.

The in vitro proliferation ability of HRMECs was measured with an 5-Ethynyl-2′-deoxyuridine Apollo 567 In Vitro Kit (Solarbio) following the manufacturer’s protocol. First, the HRMECs were incubated with 50 μmol/l 5-Ethynyl-2′-deoxyuridine at 37°C for 2 hours. Then, the cells were fixed with 4% paraformaldehyde for 30 minutes and incubated with glycine for 5 minutes. Next, the cells were rinsed first with 0.5% Triton X-100 in PBS for 10 minutes and then incubated with an Apollo reaction cocktail for 30 minutes in the dark. Hoechst was utilized to stain nucleic acids.

### RNA pull-down.

Biotin-labeled ssRNA probes were synthesized in vitro (Sangon Biotin). The oligonucleotide sequences of the probes are provided in Supplemental Table 4. An in vitro RNA protein pull-down assay was carried out using a Pierce Magnetic RNA-Protein Pull-Down Kit (Thermo Fisher Scientific) according to the manufacturer’s instructions. One hundred picomoles of RNA and 50 μL of magnetic beads were used per sample. The input RNA of each sample was mixed with 1 μL of 50% glycerol, separated on an 8% native TBE gel, and visualized by phosphor imaging using a Personal Molecular Imager (Bio-Rad).

### Luciferase reporter assay.

The sequences of *TNIP1* 3′-UTR were amplified and subcloned to the pmirGLO plasmids (Promega) and were termed wild-type–*TNIP1*. The sequences of the 8 mutant *TNIP1* 3′-UTR were amplified and subcloned to the pmirGLO plasmids (Promega) and were termed MT (1~8)-*TNIP1* (Figure 7D). Cells were cotransfected with each of these firefly luciferase reporter constructs and the Renilla luciferase control vector pGL4.73 (Promega). Twenty-four hours after transfection, firefly and Renilla luciferase signals were quantified using a Dual-Glo Luciferase Assay System (E2920, Promega) in a GloMax 96 Microplate Luminometer (E6521, Promega) according to the manufacturer’s instructions.

### RNA-binding protein immunoprecipitation–qPCR.

The RNA-binding protein immunoprecipitation (RIP) assay was performed using an Imprint RNA Immunoprecipitation Kit (RIP-12RXN, Sigma-Aldrich) according to the manufacturer’s instructions. Briefly, the corresponding cell lysates were incubated with beads coated with 5 μg control IgG antibody and anti-m^6^A antibody with rotation at 4°C overnight. Next, total RNA was extracted for the detection of *TNIP1* expression by qRT-PCR.

### RNA half-life detection.

Approximately 5 × 10^5^ HRMECs were seeded per well in 6-well plates. Two days later, actinomycin-D (10 μg/mL, Sigma-Aldrich) was administered to the cells, and the RNA was collected at 3, 6, 9, and 12 hours for qRT-PCR analysis. The half-life of the RNA was calculated according to the following equation: ln (C*i*/C0) = –*kti*, where Ci is the mRNA value at time *i*, *ti* is the time interval in hours, and *k* is the degradation rate.

### RNA extraction, library construction, Illumina sequencing (RNA-Seq), and data analysis.

Total RNA was extracted from wild-type murine retinas and their diabetic littermates by TRIzol reagent (Invitrogen). RNA integrity was assessed using the RNA Nano 6000 Assay Kit of the Bioanalyzer 2100 system (Agilent Technologies). A total concentration of 1 μg RNA per sample was used as an input for the RNA sample preparations. Clustering of the index-coded samples was performed on a cBot Cluster Generation System using TruSeq PE Cluster Kit v3-cBot-HS (Illumina) according to the manufacturer’s instructions. After cluster generation, the library preparations were sequenced on an Illumina NovaSeq 6000 platform, and 150 bp paired-end reads were generated.

Differential expression analysis of the 2 groups was performed using the DESeq2 R package (version 1.20.0). DESeq2 provides statistical routines for determining differential expression in digital gene expression data using a model based on the negative binomial distribution. The resulting *P* values were adjusted using Benjamini and Hochberg’s approach for controlling the false discovery rate. Transcripts with a fold change cutoff of more than 1 or less than –1 and a corrected *P* value cutoff of less than 0.05 were considered significantly differentially expressed genes.

GO enrichment analysis of the differentially expressed genes was conducted by the clusterProfiler R package. GO terms with a corrected *P* value of less than 0.05 were considered significantly enriched by differentially expressed genes.

### MeRIP-Seq and data analysis.

MeRIP-Seq was carried out by Novogene. Briefly, a total of 300 μg RNA was extracted from the retinas of both wild-type rats and their diabetic littermates. The integrity and concentration of extracted RNAs were determined using an Agilent 2100 bioanalyzer and simpliNano spectrophotometer (GE Healthcare), respectively. Fragmented RNA (~100 nt) was incubated for 2 hours at 4°C with an anti-m^6^A polyclonal antibody for the immunoprecipitation experiment. Then, immunoprecipitated RNA or input was used for library construction with the Ovation SoLo RNA-Seq System Core Kit (NuGEN). The library preparations were sequenced on an Illumina NovaSeq 6000 platform with a paired-end read length of 150 bp according to standard protocols. Sequencing was carried out with 3 independent biological replicates.

After mapping reads to the reference genome, the exome Peak R package (version 2.16.0) was used for m^6^A peak identification in each anti-m^6^A immunoprecipitation group with the corresponding input samples serving as a control, and a *q* value threshold of enrichment of 0.05 was used for all data sets. The m^6^A-enriched motifs of each group were identified by HOMER (version 4.9.1). In the peak calling result, each peak corresponded to a gene in which the peak was located in its exon. These genes were considered peak-related genes. In addition, the distribution of peaks on different functional regions, such as the 5′-UTR, CDS, and 3′-UTR, was determined.

Differential peak calling was performed using the exome Peak R package (version 2.16.0), with parameters including a *P* value of less then 0.05 and fold change of more than 1. Using the same method, genes associated with different peaks were identified, and GO enrichment analysis was performed.

### Statistics.

All data were analyzed using GraphPad Prism 8.0 software and SPSS (version 26.0). First, data were tested for normality by the Pearson normality test. Mean ± SD and median (interquartile range) were reported for the description of categorical variables and continuous variables with normal and skewed distribution, respectively. Means were compared using unpaired 2-tailed Student’s *t* test (2-group comparisons) and 1-way ANOVA followed by Bonferroni’s test (multigroup comparisons). Medians were compared with the nonparametric Mann-Whitney *U* test (2-group comparisons) and Kruskal-Wallis’s test followed by Bonferroni’s test (multigroup comparisons). In addition, 2-way ANOVA was applied when 2 independent variables were included. Best-corrected visual acuity was converted to the logarithm of the minimum angle of resolution (logMAR). Finger counting, hand motion, and light perception were assigned logMAR units of 2.1, 2.4, and 2.7, respectively. A *P* value of less than 0.05 was considered statistically significant.

### Study approval.

A total of 30 patients with proliferative diabetic retinopathy were recruited at Shanghai General Hospital from May 2022 to January 2023. Another 30 patients with idiopathic epiretinal membrane were selected to act as controls. Written informed consent was obtained from all patients. This study was approved by the institutional research ethics committee of Shanghai General Hospital, Shanghai Jiao Tong University School of Medicine (no. 2019SQ221, 2022KY024). The mice were maintained according to the *Guide for the Care and Use of Laboratory Animals* (National Academies Press, 2011), and they were handled according to the guidelines of the Statement for the Use of Animals in Ophthalmic and Vision Research. The experiments were approved by the Animal Care and Use Committee of Shanghai General Hospital.

### Data availability.

Values for all data points in graphs are reported in the Supporting Data Values file. The data are available from the corresponding author on reasonable request. See complete unedited blots in the supplemental material.

## Author contributions

ZZ, XX, and HC designed the research. CZ, XS, CG, YH, and MM performed experiments and/or analyzed data. QQ and TS provided advice on experiments. XX, HC, and ZZ obtained funding and supervised the research. CZ, XS, and CG wrote the paper. CZ, XS, and CG are co–first authors; authorship order reflects the degree to which authors drove key developments in the work.

## Supplementary Material

Supplemental data

Supporting data values

## Version 1 10/02/2023

Electronic publication
