# Supplementary material for: FTO fuels diabetes-induced vascular endothelial dysfunction associated with inflammation by erasing m6A methylation of TNIP1
Source: J Clin Invest. 2023 Oct 2;133(19):e160517. doi: 10.1172/JCI160517 (PMC10541204; doi:10.1172/JCI160517)

SUPPLEMENTAL MATERIAL

For paper entitled "**FTO fuels diabetes-induced vascular endothelial dysfunction associated with inflammation by erasing m^{6}A methylation of *TNIP1***"

Chuandi Zhou[1#], Xinping She[1#], Chufeng Gu[1#], Yanan Hu[1], Mingming Ma[1], Qinghua Qiu[1], Tao Sun[2], Xun Xu[1*], Haibing Chen[3*], Zhi Zheng[1*].

Authors: Chuandi Zhou[1#], Xinping She[1# †], Chufeng Gu[1#], Yanan Hu[1], Mingming Ma[1], Qinghua Qiu[1§], Tao Sun[2], Xun Xu[1*], Haibing Chen[3*], Zhi Zheng[1*]

Institutional affiliations:

[1]Department of Ophthalmology, Shanghai General Hospital, Shanghai Jiao Tong University School of Medicine; National Clinical Research Center for Eye Diseases; Shanghai Key Laboratory of Ocular Fundus Diseases; Shanghai Engineering Center for Visual Science and Photomedicine; Shanghai engineering center for precise diagnosis and treatment of eye diseases.

[2]Shanghai Eye Diseases Prevention & Treatment Center Shanghai Eye Hospital; Shanghai General Hospital; National Clinical Research Center for Eye Diseases; Shanghai Key Laboratory of Ocular Fundus Diseases.

[3]Department of Endocrinology and Metabolism, Shanghai 10th People's Hospital, School of Medicine, Tongji University

[†]Current Affiliation: Division of Ophthalmology, The Affiliated Hospital of Hangzhou Normal University

[§]Current Affiliation: Department of Ophthalmology, Tong Ren Hospital, Shanghai Jiao Tong University School of Medicine

[#]Chuandi Zhou, Xinping She and Chufeng Gu contributed equally to this paper.

[*]Zhi Zheng, Xun Xu and Haibing Chen are corresponding authors.

Supplemental material consisted of 11 supplemental figures and 6 supplemental tables.

Supplemental Figure 1

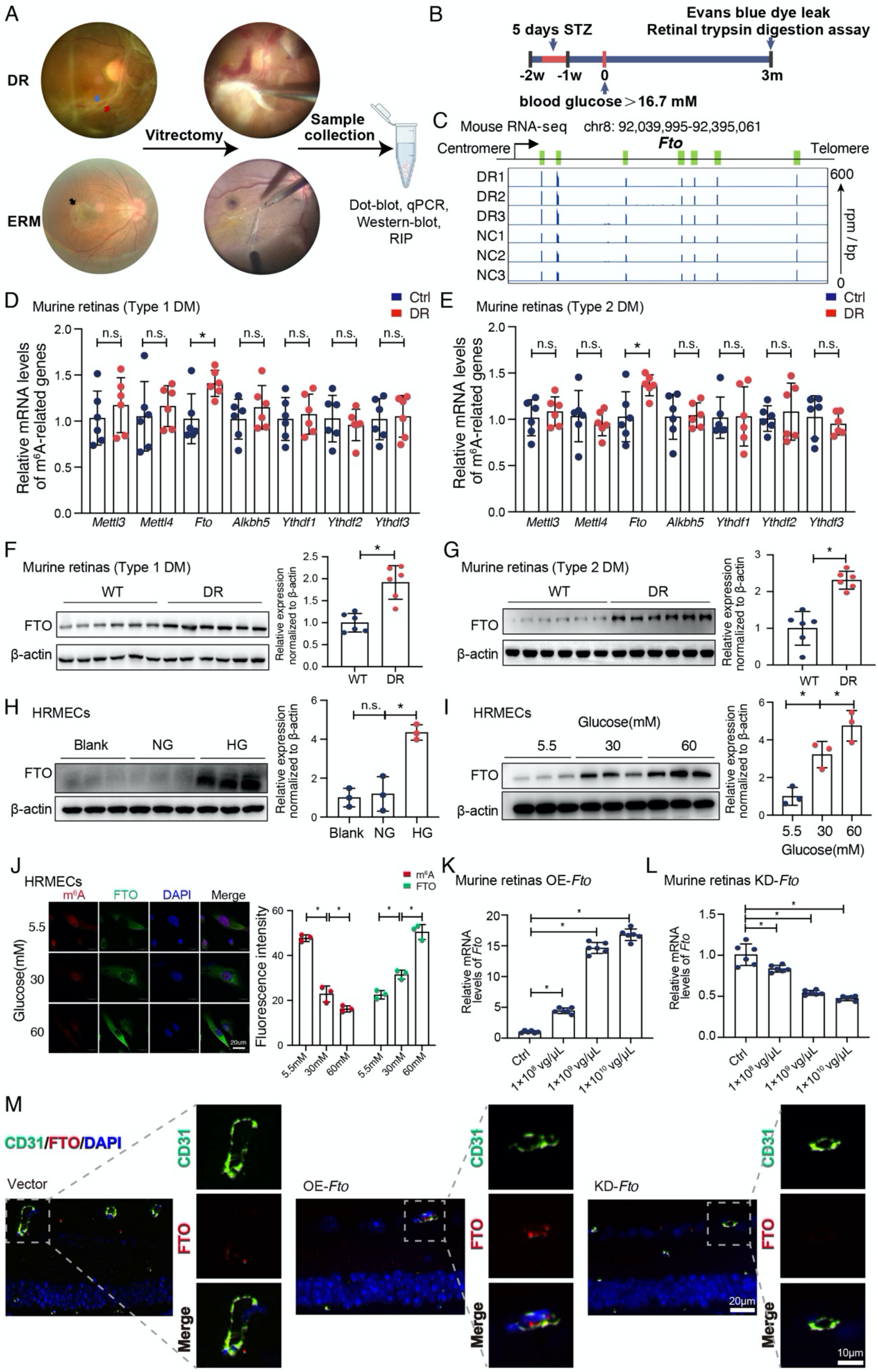

Supplemental Figure 1. FTO is elevated upon high glucose both in vitro and in vivo.

(A) A diagram showing patient sample collection. Representative fudus photographs of recruited patients with diabetic retinopathy (DR) and idiopathic epiretinal membrane (ERM) (preoperative: the first column; intraoperative: the second column). Blue arrow: extensive fibrovascular membranes; Red arrow: neovascularization on fibrovascular membranes; Black arrow: epiretinal membrane formed on macular. (B) A schematic diagram of induction of diabetes and phenotype experiments in mice. (C) Gene tracks based on RNA-seq of *Fto* using Integrative Genomics Viewer (IGV) in normal and diabetic mouse retinas. DR, diabetic retinopathy; rpm/bp, reads per million mapped reads per base pair. (D and E) qRT-PCR reveals higher levels of *FTO* in murine retinas with type 1 (D, n=6) or type 2 (E, n=6) diabetes (Mann-Whitney U test). (F and G) Western blotting shows elevated expression of FTO in murine retinas with type 1 (F, n=6) or type 2 (G, n=6) diabetes (Student's t test). (H) Western-blotting shows upregulated FTO treated by high glucose in human retinal microvascular endothelial cells (HRMECs) (n=3). NG, normal glucose (5.5 mM) with D-mannitol as osmotic control; HG, high glucose (30 mM). (I) Western-blotting indicates FTO expression is elevated with increasing concentrations of glucose administered to HRMECs (n=3). (J) Immunofluorescence shows reduced level of m^6A and upregulated FTO in HRMECs treated with increasing concentrations of glucose (n=3, scale bar: 20 μm). (K, L) qRT-PCR shows overexpression (K, n=6) and knockout (L, n=6) efficiency of *Fto* by intravitreal injection of adeno-associated virus (AAV). (M) Immunofluorescence shows successful transfection of AAV in retinal vascular endothelial cells to modulate the expression of FTO (scale bar: 10 μm; zoom figure: scale bar: 20 μm). For H-L, significant differences are determined by Kruskal-Wallis's test followed by Bonferroni's post hoc comparison test. Data are shown as the mean ± SD. *$p<0.05$. n.s., statistically not significant.

Supplemental Figure 2

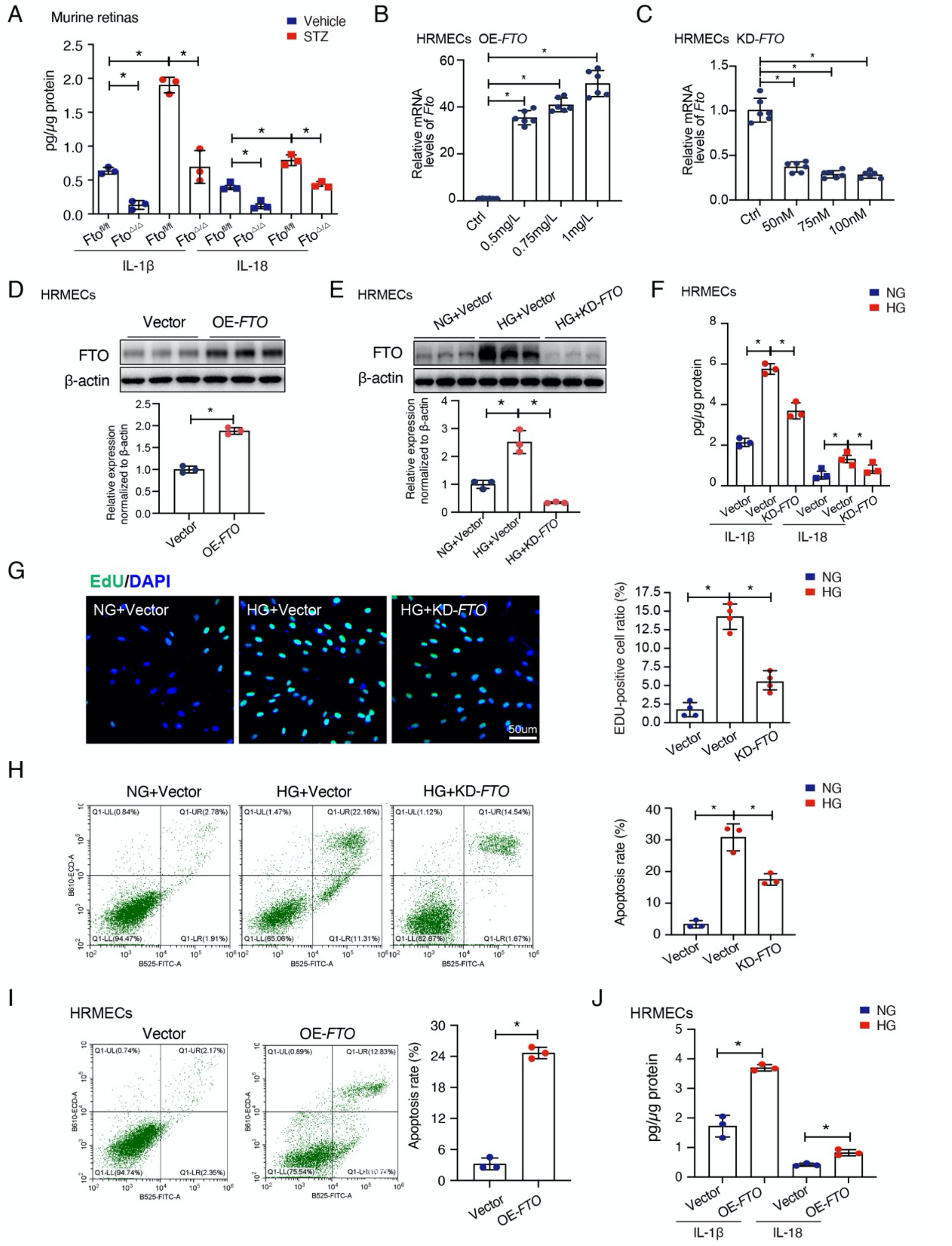

Supplemental Figure 2. FTO promotes inflammation, proliferation and apoptosis upon high glucose both *in vivo* and *in vitro*.

(A) ELISA assays demonstrate EC-specific *Fto*-deficient (EC $Fto^{\Delta/\Delta}$) mice have lower levels of interleukin (IL)-1β and IL-18 in retina after introduction of diabetes (n=3). (B, C) qRT-PCR shows overexpression (B, n=6) and knockout efficiency (C, n=6) of *FTO* by overexpression plasmid and small interfering RNA (siRNA), respectively. (D) Western-blotting shows upregulated FTO in human retinal microvascular endothelial cells (HRMECs) after transfecting with overexpressed

plasmid (n=3, Student's t test). (E) Western-blotting displays upregulated FTO upon high glucose in HRMECs, and FTO is successfully knockout by siRNA (n=3). (F) ELISA assays display an elevation of IL-1β and IL-18 in HRMECs treated by high glucose, and this trend can be reversed by silencing *FTO* (n=3). (G) EdU assays indicates silencing *FTO* suppressed the proliferation of HRMECs induced by high glucose. The nuclei were stained with Hoechst (blue) (n=4, Scale bar: 50 μm). (H) Flow cytometric analysis demonstrates *FTO* knockdown alleviates apoptosis of HRMECs treated by high glucose (n=3). (I) Flow cytometric analysis indicates *FTO* overexpression aggravates apoptosis of HRMECs (n=3, Student's t test). (J) ELISA assays display overexpressed *FTO* lead to an elevation of IL-1β and IL-18 (n=3, Student's t test). NG, normal glucose (5.5 mM) with D-mannitol as osmotic control; HG, high glucose (30 mM). For A-C, E-H, significant differences are assessed by 1-way ANOVA or Kruskal-Wallis's test followed by Bonferroni's post hoc comparison test. Data are shown as the mean ± SD. $*p<0.05$.

Supplemental Figure 3

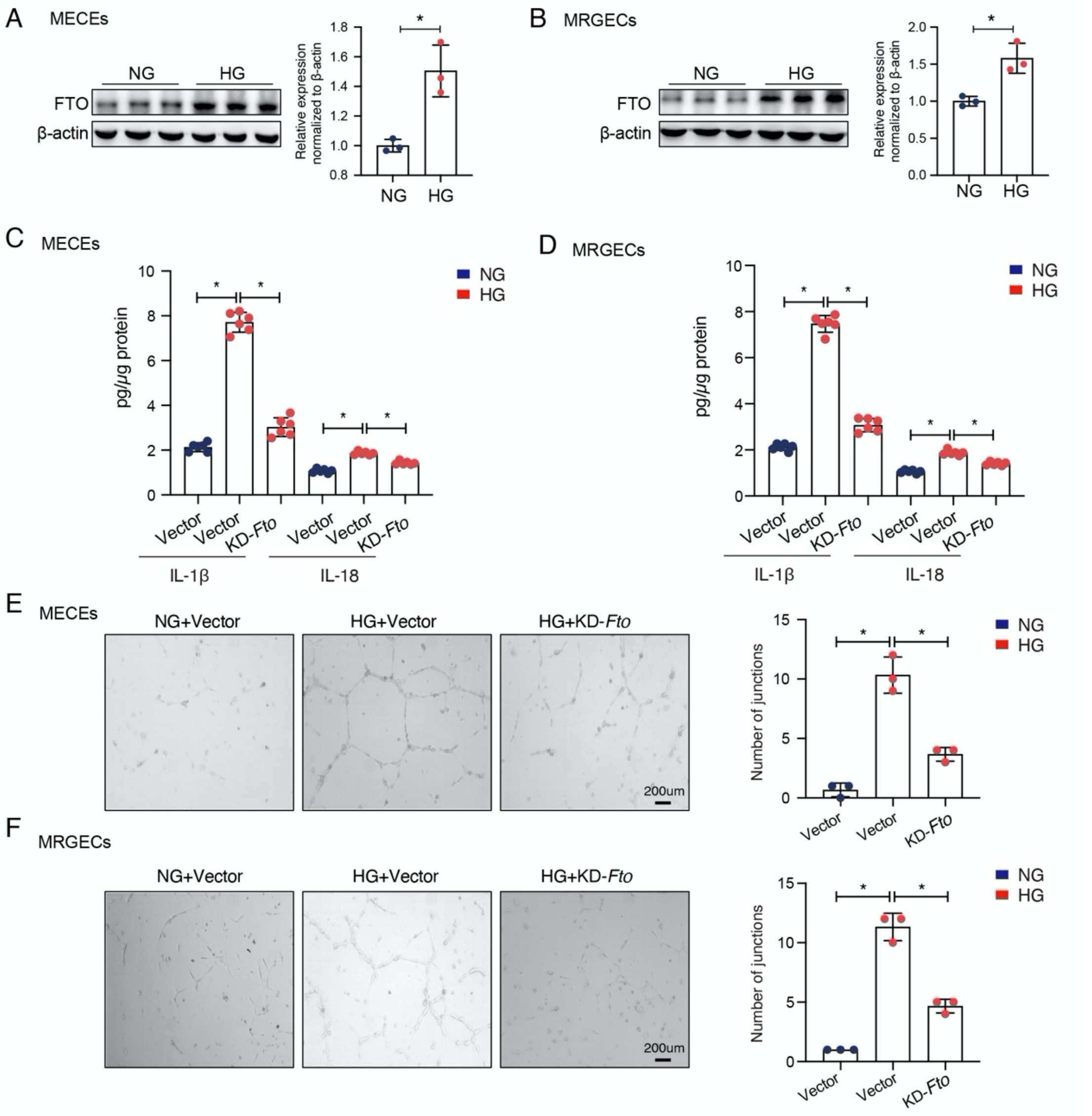

Supplemental Figure 3. Silencing *Fto* inhibits inflammation and tube formation induced by high glucose in both mice cardiac endothelial cells (MECEs) and mice renal glomerular endothelial cells (MRGECs).

(A, B) Western-blotting displays upregulated FTO upon high glucose in both MECEs (A, n=3, Student's t test) and MRGECs (B, n=3, Student's t test). (C, D) ELISA assays show silencing *Fto* suppresses elevated interleukin-1β (IL-1β) and IL-18 stressed by high glucose in both MECEs (C, n=6) and MRGECs (D, n=6). (E, F) Silencing *Fto* inhibits tube formation induced by high glucose in both MECEs (E, n=3, scale bar: 200 μm) and MRGECs (F, n=3, scale bar: 200 μm). The average number of tube formation for each field was assessed. NG, normal glucose (5.5 mM) with D-mannitol as osmotic control; HG, high glucose (30 mM). For C-F, significant differences are determined by 1-way ANOVA or Kruskal-Wallis's test followed by Bonferroni's post hoc comparison test. Data are shown as the mean ± SD. *p<0.05. n.s., statistically not significant.

Supplemental Figure 4

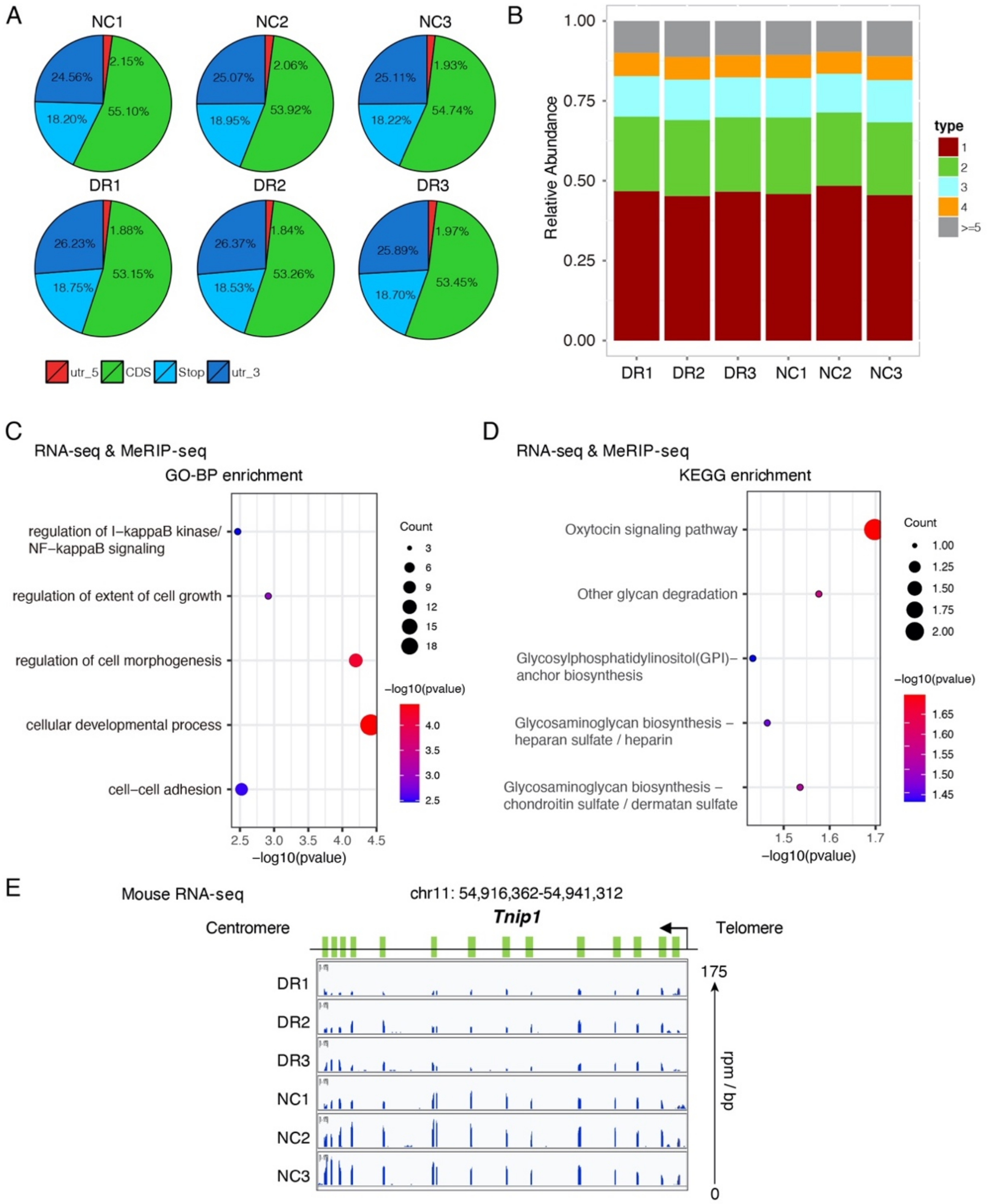

Supplemental Figure 4. Enrichment analyses of differentially expressed genes in diabetes. (A) Pie charts exhibit the distribution of m^{6}A peak in different RNA regions (CDS, 5′ UTR, 3′ UTR and stop codon) in normal and diabetic murine retinas. (B) The distribution of the genes with 1, 2, 3, 4 and 5 or more m^{6}A peaks in normal and diabetic murine retinas. (C) Gene ontology (GO) analysis for differentially expressed genes in diabetic reitnas based on combined RNA-seq and MeRIP-seq. (D) Kyoto Encyclopedia of Genes and Genomes (KEGG) analysis for differentially expressed genes in diabetic reitnas based on combined RNA-seq and MeRIP-seq. DR, diabetic retinopathy. (E) Gene tracks based on RNA-seq of *Tnip1* using Integrative Genomics Viewer (IGV) in normal and diabetic mouse retinas. DR, diabetic retinopathy; rpm/bp, reads per million mapped reads per base pair.

Supplemental Figure 5

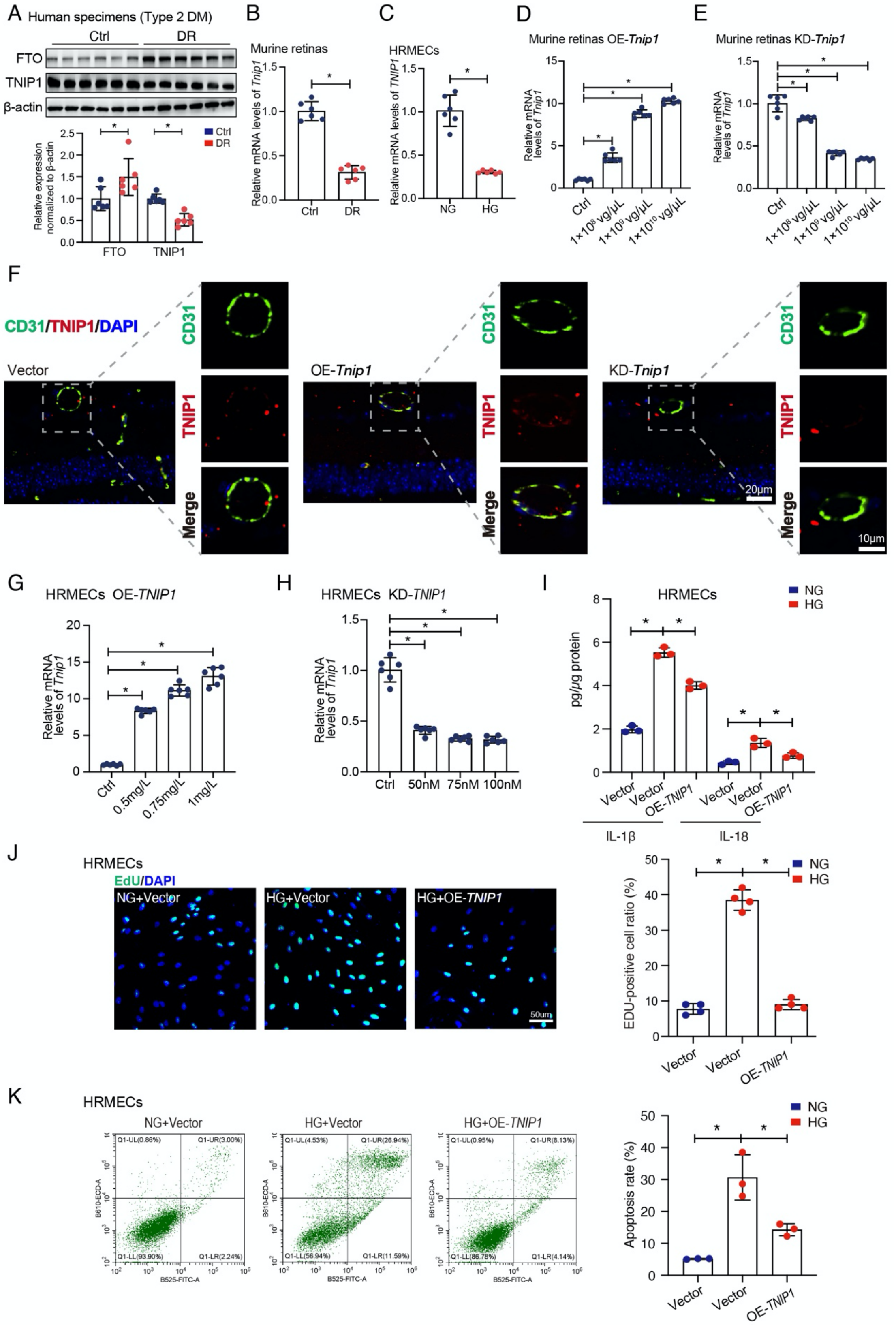

Supplemental Figure 5. ***TNIP1*** **attenuates inflammation, proliferation and apoptosis stressed by high glucose.**

(A) Western blotting displays reduced expression of TNIP1, accompanied by upregulated FTO, in retinal fibrovascular membranes of patients with retinopathy due to type 2 diabetes (n=6, Student's t test). (B, C) qRT-PCR shows suppressed expression of *Tnip1* in both diabetic murine retinas (B, n=6, Student's t test) and human retinal microvascular endothelial cells (HRMECs) cultivated in high glucose (C, n=6, Student's t test). NG, normal glucose (5.5 mM) with D-mannitol as osmotic control; HG, high glucose (30 mM). (D, E) qRT-PCR shows overexpression (D, n=6) and knockout (E, n=6) efficiency of *Tnip1* by intravitreal injection of adeno-associated virus (AAV). (F) Immunofluorescence shows successful transfection of AAV in retinal vascular endothelial cells to modulate the expression *Tnip1* (scale bar: 10 μm; zoom figure: scale bar: 20 μm). (G, H) qRT-PCR shows overexpression (G, n=6) and knockout efficiency (H, n=6) of *Tnip*1 by overexpression plasmid and small interfering RNA (siRNA), respectively. (I) ELISA assays demonstrate overexpressed *TNIP1* suppresses the enrichment of interleukin-1β (IL-1β) and IL-18 in human retinal microvascular endothelial cells (HRMECs) treated by high glucose (n=3). (J) EdU assays displays *TNIP1* impedes the proliferation of HRMECs cultivated in high glucose. The nuclei were stained with Hoechst (blue). (n=4, scale bar: 50 μm). (K) Flow cytometric analysis demonstrates *TNIP1* alleviates apoptosis of HRMECs treated by high glucose (n=3). NG, normal glucose (5.5 mM) with D-mannitol as osmotic control; HG, high glucose (30 mM). For D-E, G-K significant differences are assessed by Kruskal-Wallis's test followed by Bonferroni's post hoc comparison test. Data are shown as the mean ± SD. *p<0.05.

Supplemental Figure 6

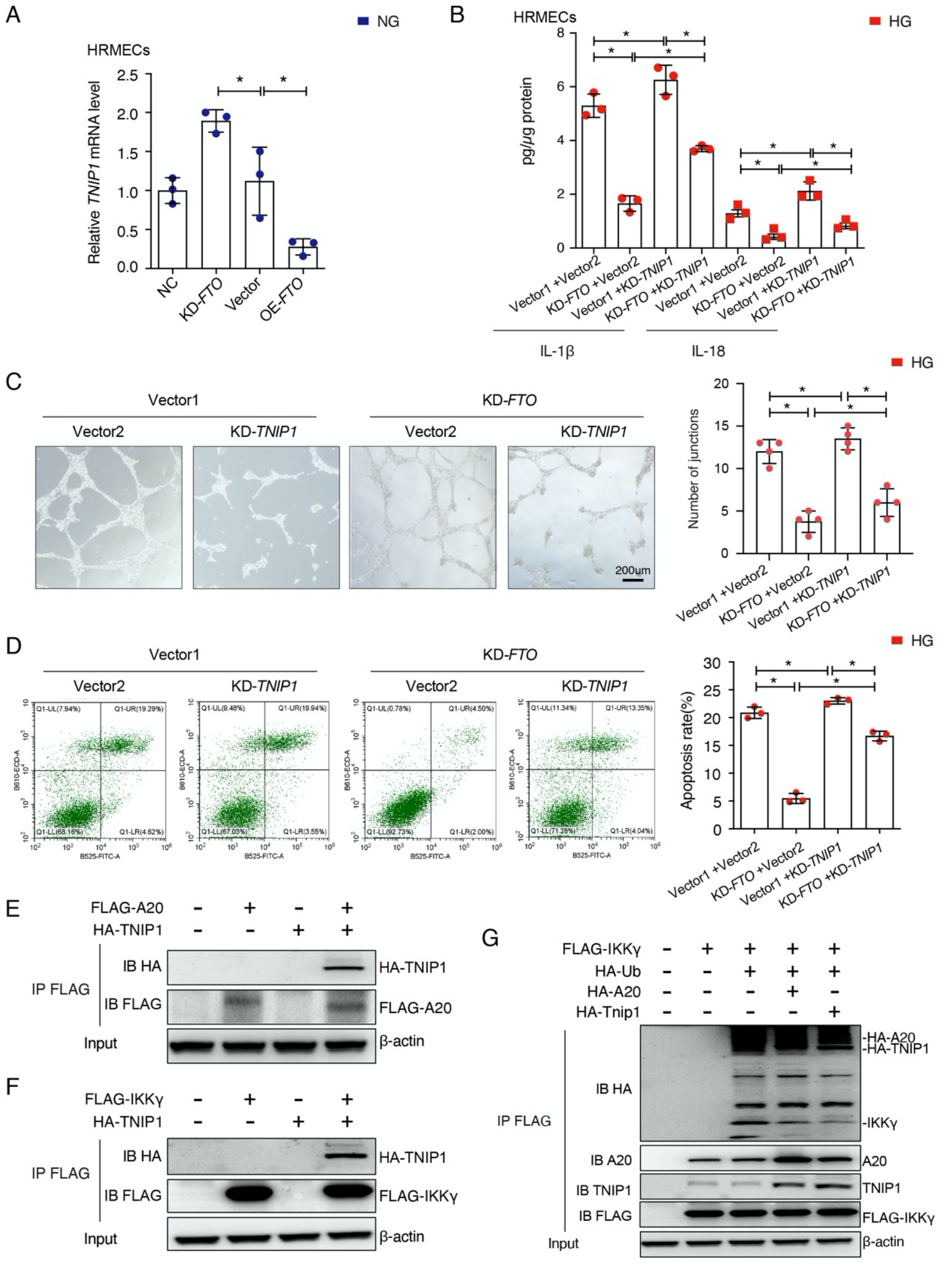

Supplemental Figure 6. FTO regulates inflammation and apoptosis in diabetes-induced retinal vascular endothelium dysfunction via mediating TNIP1.

(A) FTO reduces mRNA level of *TNIP1* in human retinal microvascular endothelial cells (HRMECs) (n=3). (B) *TNIP1* knockout partially reverses the trend of decreased interleukin-1β (IL-1β) and IL-18 caused by silencing *FTO* in HRMECs (n=3). (C) Silencing *TNIP1* limits the suppression effect of tube formation induced by *FTO* knockdown. The average number of tube formation for each field

was assessed (n=4, scale bar: 200 μm). (D) Silencing *TNIP1* partially reverses the trend of reduced apoptosis induced by *FTO* knockout in HRMECs (n=3). (E) Co-immunoprecipitation (Co-IP) assays reveal direct interaction between TNIP1 and A20. (F) Co-IP assays identify direct interaction between TNIP1 and IKKγ. (G) Co-IP assays indicate TNIP1 promotes A20-mediated deubiquitination of IKKγ. NG, normal glucose (5.5 mM) with D-mannitol as osmotic control; HG, high glucose (30 mM). All significant differences are assessed by 1-way ANOVA or Kruskal-Wallis's test followed by Bonferroni's post hoc comparison test. Data are shown as the mean ± SD. $^*p<0.05$.

Supplemental Figure 7

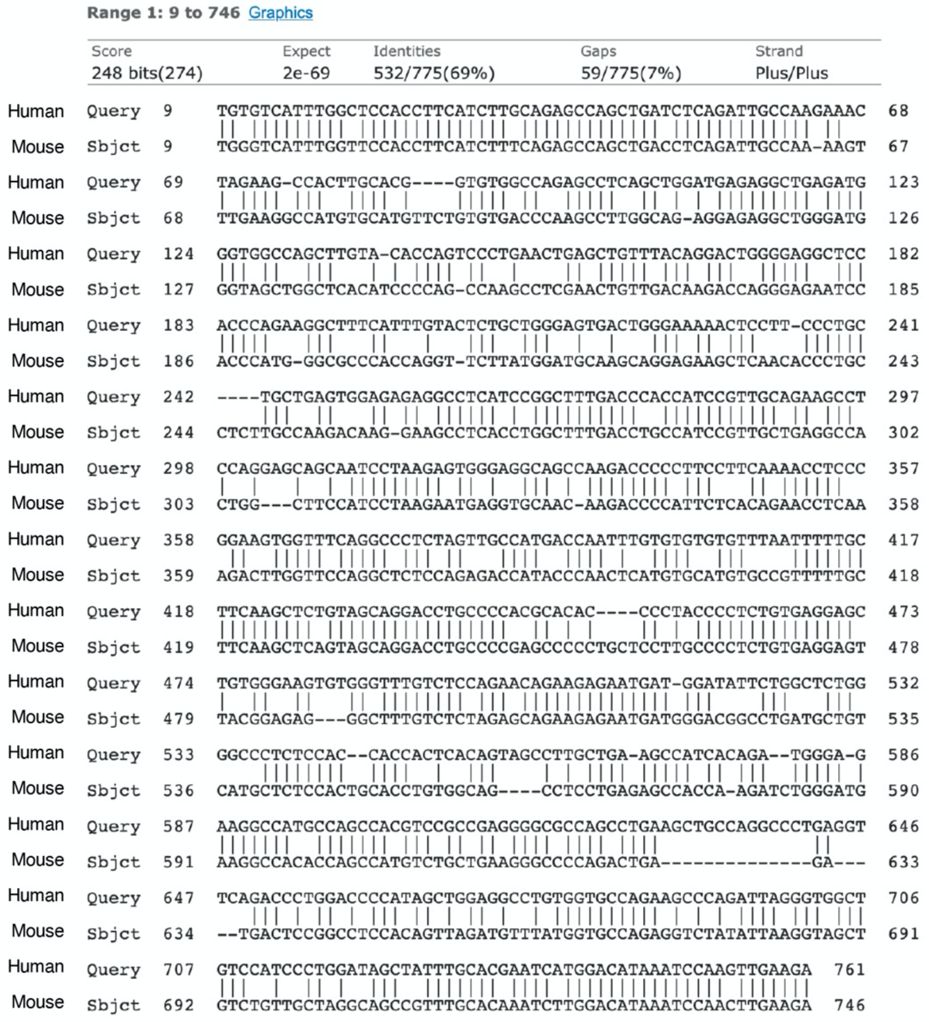

Supplemental Figure 7. *TNIP1* gene homology comparison between human and mouse. *TNIP1*homology analysis with Basic Local Alignment Search Tool (BLAST) between human and mouse genomes.

Supplemental Figure 8

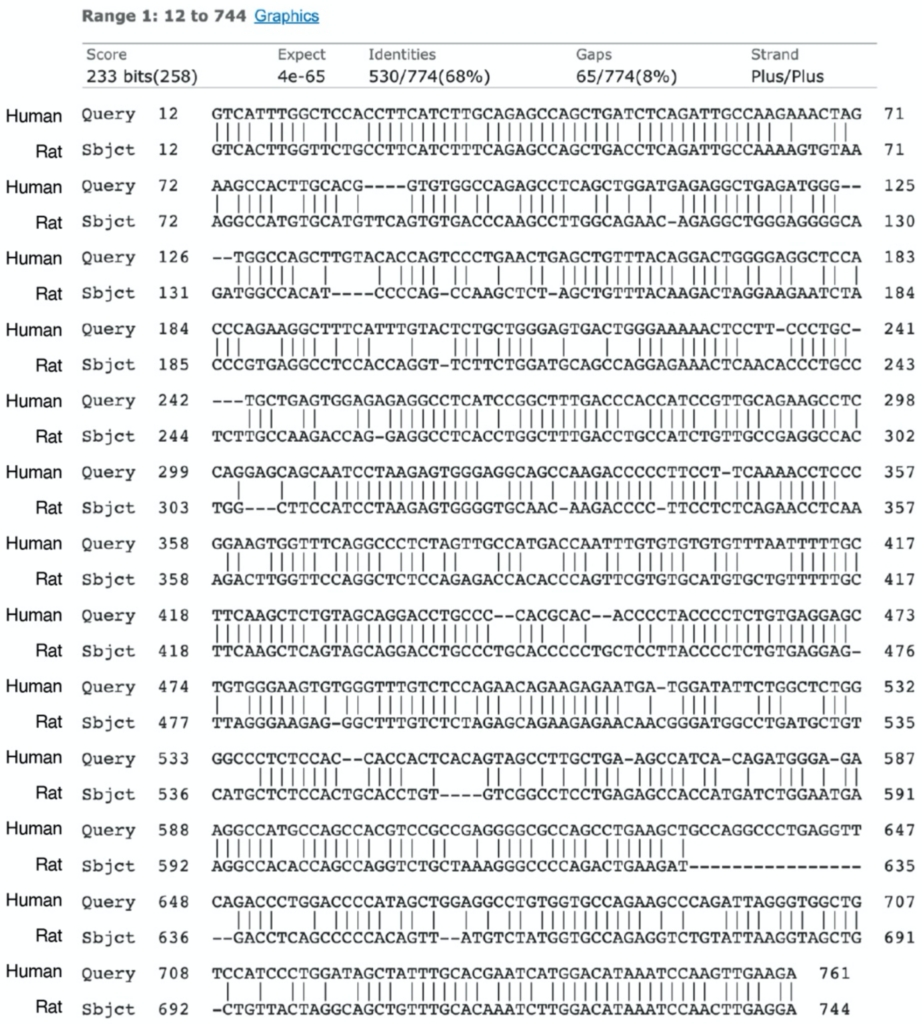

Supplemental Figure 8. *TNIP1*gene homology comparison between human and rat. ***TNIP1***homology analysis with Basic Local Alignment Search Tool (BLAST) between human and rat genomes.

Supplemental Figure 9

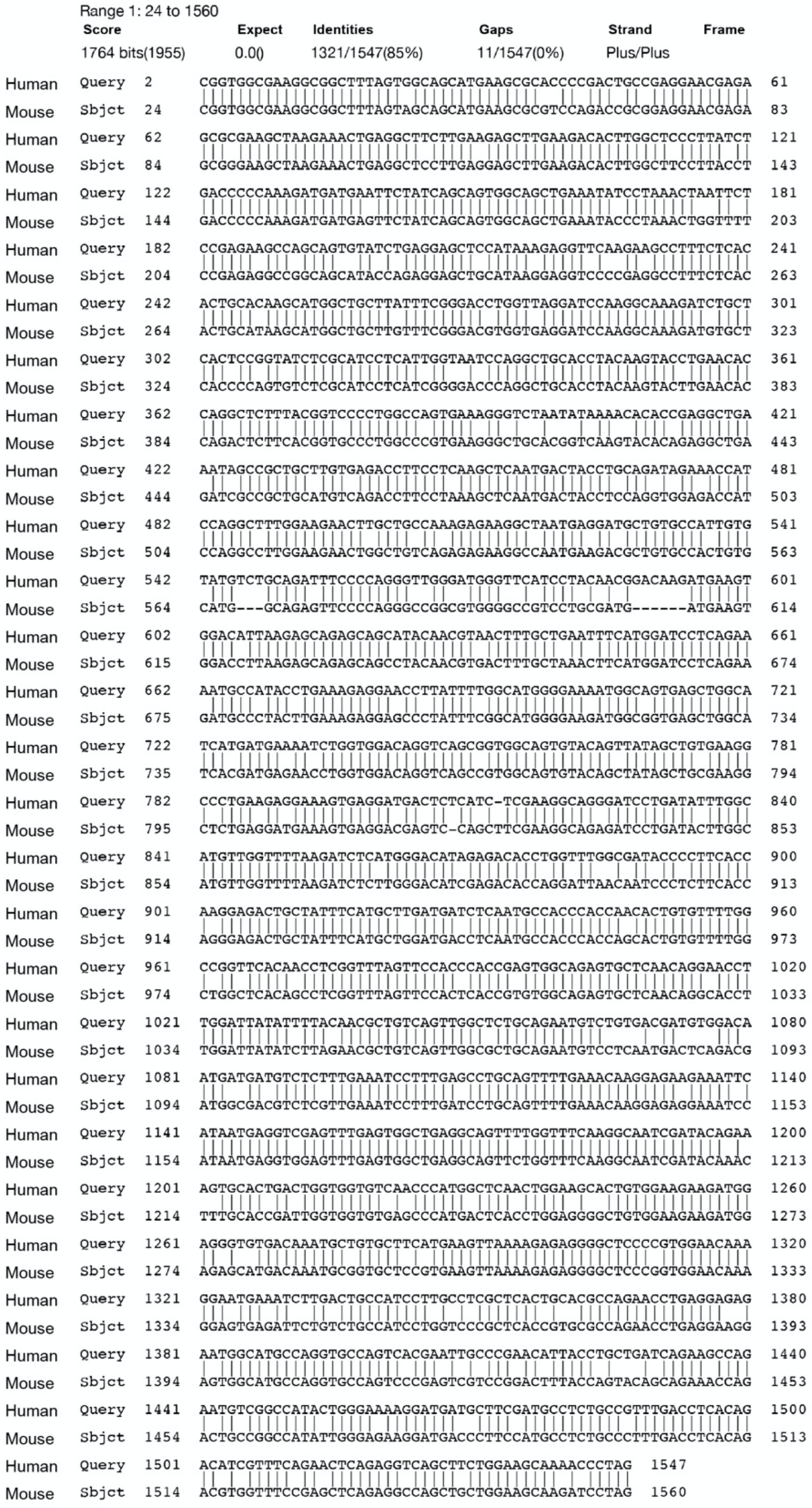

Supplemental Figure 9. *FTO* gene homology comparison between human and mouse.

FTO homology analysis with Basic Local Alignment Search Tool (BLAST) between human and mouse genomes.

Supplemental Figure 10

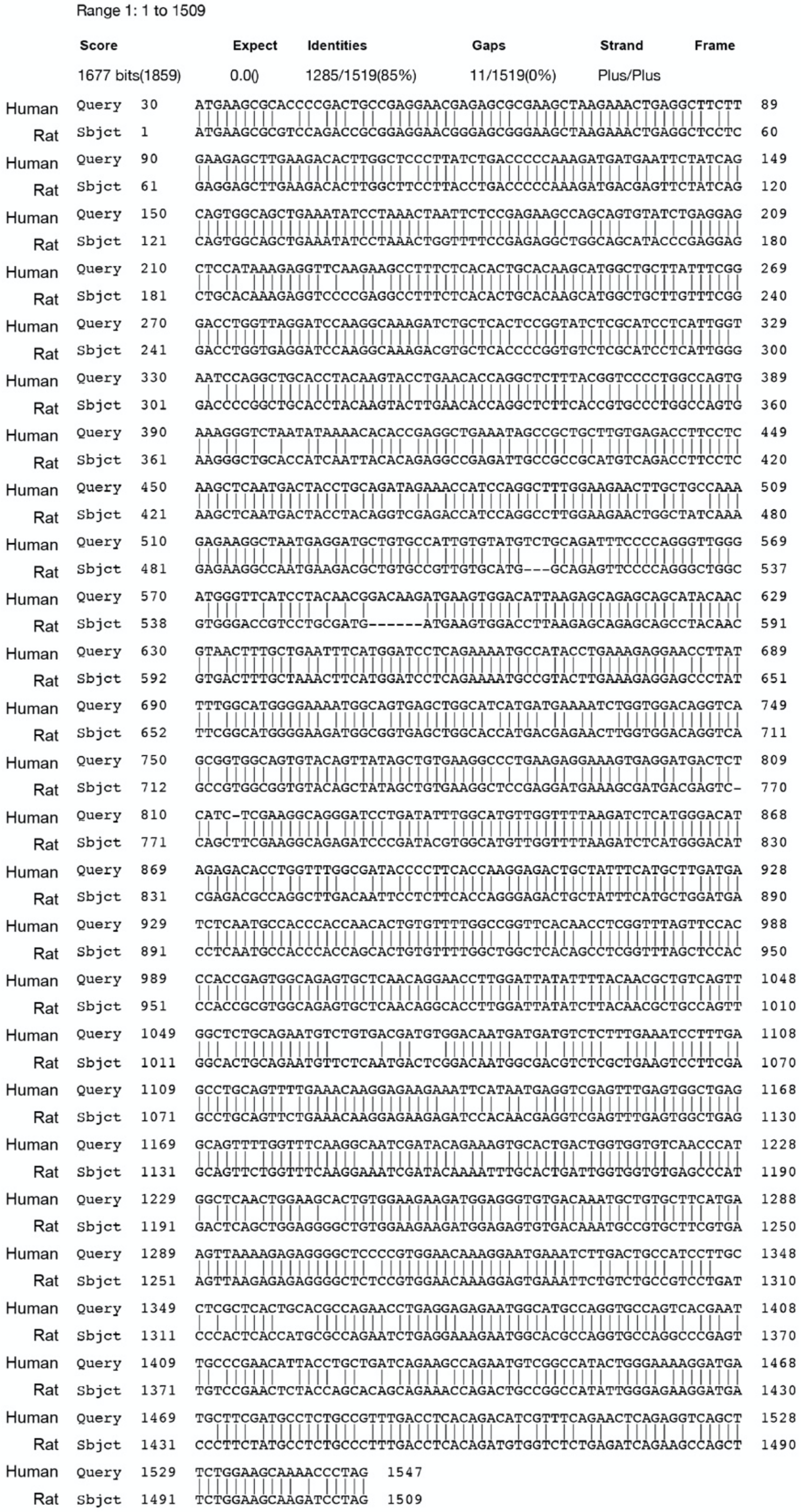

Supplemental Figure 10. ***FTO*** **gene homology comparison between human and rat.**

FTO homology analysis with Basic Local Alignment Search Tool (BLAST) between human and rat genomes.

Supplemental Figure 11

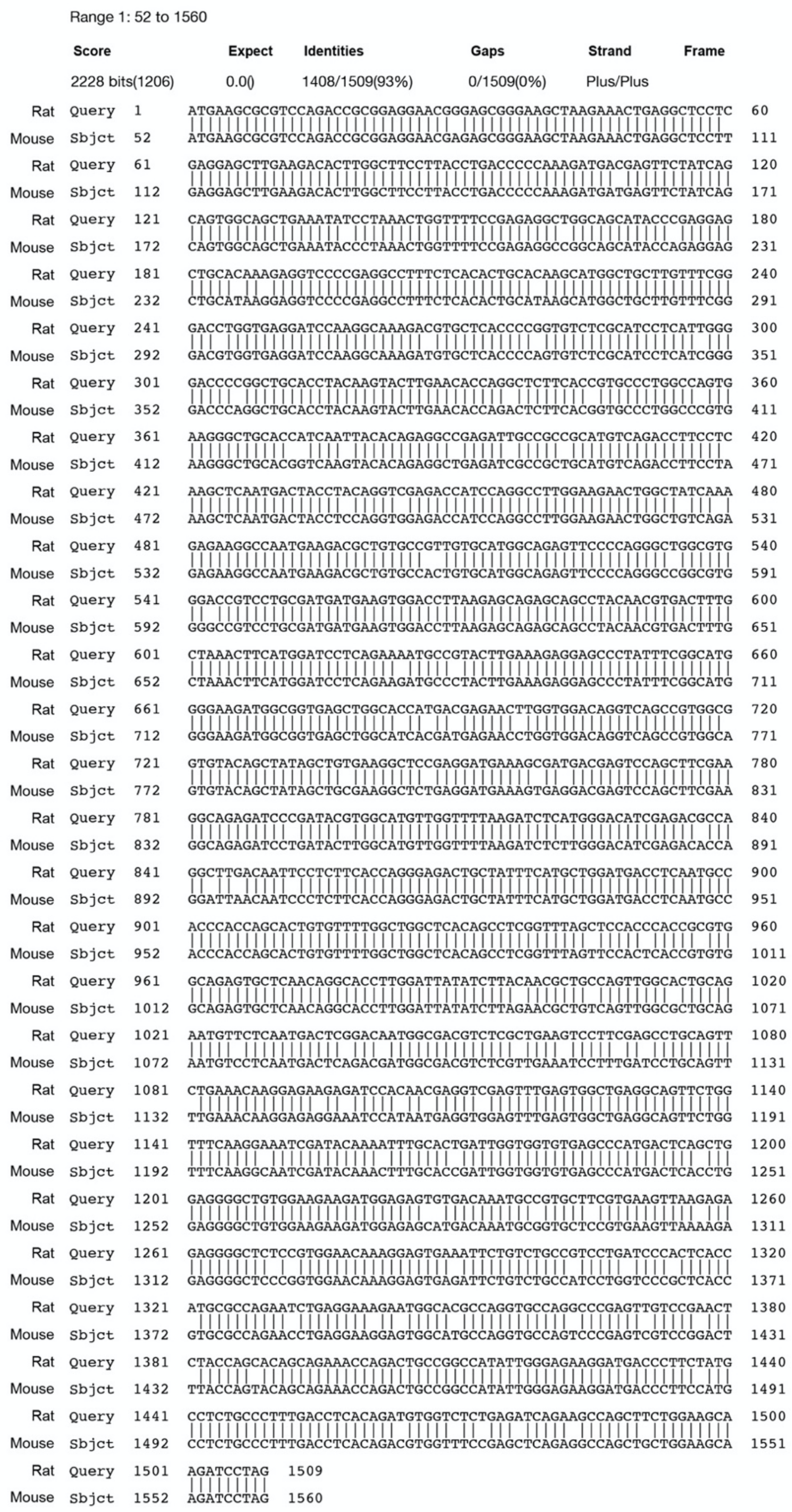

Supplemental Figure 11. *Fto* gene homology comparison between rat and mouse.
Fto homology analysis with Basic Local Alignment Search Tool (BLAST) between rat and mouse genomes.

Supplemental Table 1. Differentially expressed genes based on combined methylated RNA immunoprecipitation sequencing (MeRIP-seq) and RNA-seq in diabetes

Gene_ID	Gene_Name	m^6A log_2 fold change	RNA log_2 fold change
ENSRNOG00000010370	*Tnip1*	-2.403344934	-1.877649774
ENSRNOG00000006565	*Fstl4*	-2.116491664	0.933593991
ENSRNOG00000001335	*Zkscan1*	-1.501391236	0.676768574
ENSRNOG00000008869	*Ppp1r9a*	-1.035297836	0.703270304
ENSRNOG00000024711	*Sdk2*	-0.926931718	0.755600279
ENSRNOG00000009403	*Dcaf17*	-0.890179084	0.831494151
ENSRNOG00000043102	*Bahcc1*	-0.786528769	0.628833773
ENSRNOG00000013570	*Rad54l2*	-0.77675958	0.623855184
ENSRNOG00000006050	*Med19*	-0.74638501	-0.593321028
ENSRNOG00000014452	*Zfhx3*	-0.733215313	0.700445655
ENSRNOG00000007528	*Kcnh7*	-0.728463539	1.240004352
ENSRNOG00000013257	*Hecw2*	-0.727604057	0.872840955
ENSRNOG00000048174	*Uqcrq*	-0.720206759	-0.684999835
ENSRNOG00000016046	*Hecw1*	-0.670929796	0.663801235
ENSRNOG00000004269	*Myt1l*	-0.657111855	0.629508064
ENSRNOG00000015269	*Atf7*	-0.630214394	0.604309124
ENSRNOG00000014866	*Pign*	-0.628593726	0.724600424
ENSRNOG00000007319	*Trib3*	-0.60789811	0.898044
ENSRNOG00000030352	*Pcdhga9*	-0.599882549	0.745519586
ENSRNOG00000007030	*Epha7*	0.607385619	0.704355376
ENSRNOG00000003742	*Cdkl5*	0.614003318	0.882585524
ENSRNOG00000052894	*Epg5*	0.624900984	0.677217193
ENSRNOG00000011585	*Fat3*	0.635607055	0.86493989
ENSRNOG00000045696	/	0.670009641	-0.720027611
ENSRNOG00000000924	*Slc7a1*	0.688531053	1.916719941
ENSRNOG00000001622	*Impg2*	0.708175115	0.649446802
ENSRNOG00000001141	*Srrm4*	0.708357455	0.669713596
ENSRNOG00000029131	*Aabr07024637.1*	0.723300489	0.657155156
ENSRNOG00000002291	*Brwd3*	0.763101043	0.68355021
ENSRNOG00000011151	*Tenm4*	0.773635986	0.688185311
ENSRNOG00000002341	*Trim25*	0.77387041	0.777095165
ENSRNOG00000024832	*Gpr158*	0.843450113	0.773135615
ENSRNOG00000056658	*Xylt1*	0.868167806	0.65151631
ENSRNOG00000056656	/	0.881474402	1.058279742
ENSRNOG00000059479	*Adcy1*	0.93522769	0.587940421
ENSRNOG00000018830	*Aff3*	0.976309003	0.792163902
ENSRNOG00000008425	*Nav1*	1.038821314	0.719154598
ENSRNOG00000006943	*Aabr07072108.1*	1.090522518	-1.181784135
ENSRNOG00000010964	*Akap13*	1.099207994	0.846206833
ENSRNOG00000000478	/	1.199220997	-0.823773518
ENSRNOG00000001216	*Trpm2*	1.45803698	-0.640956169
ENSRNOG00000053240	*Soga1*	1.475450762	0.834672276
ENSRNOG00000052247	*Manba*	1.820481272	0.587818667

Supplemental Table 2. Genes regulated by *Fto* based on combined analyses of methylated RNA immunoprecipitation sequencing (MeRIP-seq) and RNA-seq in diabetic retinas

Gene_ID	Gene_name	m^6A log_2 fold change	RNA log_2 fold change
ENSRNOG00000061371	*Aabr07035541.3*	-1.501391236	-2.986475067
ENSRNOG00000022673	*Slc22a20*	-1.709196683	1.413439099
ENSRNOG00000017410	*Loxhd1*	-1.394557866	1.324649995
ENSRNOG00000010370	*Tnip1*	-2.403344934	-1.877649774
ENSRNOG00000051494	*Aabr07002870.2*	-1.599254974	4.299916654
ENSRNOG00000010906	*Ccl5*	-1.139213224	-1.258196724
ENSRNOG00000029749	*Pabpc4l*	-5.766610799	2.305523471
ENSRNOG00000015441	*Il4r*	-2.223895882	1.25294364
ENSRNOG00000028632	*Bcl2l14*	-1.029716879	1.413439099
ENSRNOG00000028744	*Mtnr1a*	-3.037795763	2.039296856
ENSRNOG00000042623	*Sh2d4b*	-1.326540478	-1.24398034
ENSRNOG00000032869	*Crygf*	-4.863984696	-2.35602882

Supplemental Table 3. Clinical characteristics of patients with diabetic retinopathy and idiopathic epiretinal membrane

Variables	DR (n=30)	ERM (n=30)	p
Age (y)	51.5(41.0-57.0)	59.0(56.5-66.0)	<0.001*
Gender			0.121
Male	18(60.0)	12(40.0)	
Female	12(40.0)	18(60.0)	
Diabetes duration (y)	12.5(10.0-17.5)	NA	NA
Type of diabetes			NA
Type 1 diabetes	10(33.3)	NA	
Type 2 diabetes	20(66.7)	NA	
Glycated hemoglobin (%)	7.1(6.4-8.6)	5.4(4.9-5.9)	<0.001*
Fasting blood glucose (mmol/L)	6.2(5.9-8.5)	5.6(4.8-6.3)	0.002*
Systolic blood pressure (mmHg)	129.5(121.5-140.3)	134.5(123.8-142.5)	0.317
Diastolic blood pressure (mmHg)	78.5(70.0-86.0)	80.0(72.8-90.0	0.552
Low density lipoprotein (mmol/L)	2.8(1.7-3.0)	2.7(2.3-2.9)	0.915
BMI (kg/m^2)	24.0(21.8-26.4)	24.8(22.7-26.4)	0.313
Insulin use	26(86.7%)	0	<0.001*
LogMAR BCVA	0.8(0.3-1.2)	0.7(0.5-1.0)	0.305

Data are presented as median (interquartile range)/n (%).

DR: diabetic retinopathy; ERM: epiretinal membrane; BMI: body mass index; LogMAR, logarithm of the minimum angle of resolution; BCVA: best-corrected visual acuity; NA: not available.

*Statistically significant.

Supplemental Table 4. Oligonucleotides sequences for small interfering RNA (siRNA), overexpression plasmid, virus, and probes

Oligonucleotides name	Species	Sequence (5'-3')
FTO-siRNA	human	CGGTGGCAGTGTACAGTTA
TNIP1-siRNA	human	CAGGAGAGCGUUACCAUGUGGTT
NC-siRNA	human	TTCTCCGAACGTGTCACGT
LV-*FTO*-OE	human	NM_001363894.1
LV-*TNIP1*-OE	human	NM_001252385.2
AAV-*Fto*-shRNA	mouse	Top strand: AATTCGTTGAAAGAGGAGCCCTATTTCCTCGAGGAAATAGGGCTCCTCTTTCAATTTTTTG<br>Bottom strand: GATCCAAAAAATTGAAAGAGGAGCCCTATTTCCTCGAGGAAATAGGGCTCCTCTTTCAACG
AAV-*Fto*-OE	mouse	NM_011936.2
AAV-*Tnip1*-shRNA	mouse	Top strand: AATTCGCGAGTTCAACAGGTTGGCCTCCAAATTCAAGAGATTTGGAGGCCAACCTGTTGAACTCGTTTTTTG<br>Bottom strand: GATCCAAAAAACGAGTTCAACAGGTTGGCCTCCAAATCTCTTGAATTTGGAGGCCAACCTGTTGAACTCGCG
AAV-*Tnip1*-OE	mouse	NM_021327.4
WT probe	/	TAAGAGTGGGAGGCAGCCAAGACCCCCTTCCTTCAAAACCTCCCGGA
MT probe	/	TAAGAGTGGGAGGCAGCCAAGTCCCCCTTCCTTCAAAACCTCCCGGA

LV: lentivirus; AAV: adeno-associated virus; OE, overexpression; WT: widetype; MT: mutant type.

Supplemental Table 5. Primers used in this study

Gene name	Application	Species	Sequence (5'-3')
TNIP1	qPCR	human	F: GTTCAACCGACTGGCATCCAA
			R: AGACGCACCCTCTTTGTTGC
METTL3	qPCR	human	F: TCCATCTGTCTTGCCATCT
			R: TCGCTTTACCTCAATCAACTC
METTL14	qPCR	human	F: AATGGCCGTTCTGTGCTCAT
			R: AAGGACCCATCACAGGCAAG
FTO	qPCR	human	F: GCTGACCTGGATGTAGATGTT
			R: GGAGAGATGTGTTAATGGCAT
ALKBH5	qPCR	human	F: CCTTGGTTTTGTTGCCTGTT
			R: ATCAGCCTCTGTCCCCTATTG
YTHDF1	qPCR	human	F: GGAACAACATCTATCAGCACA
			R: GACCTTGAGACCCACTTGTC
YTHDF2	qPCR	human	F: GCTACAAGCACACCACTTCCAT
			R: GCCTTTTATTTCCCACGACC
YTHDF3	qPCR	human	F: CCTGTCAGTGCTTCACCTTCT
			R: CGTCCATTCTTCAGATTCCAA
β-actin	qPCR	human	F: GCACCGCAAATGCTTCTA
			R: GGTCTTTACGGATGTCAACG
TNIP1	m^6A RIP	human	F: AGAGTGGGAGGCAGCCA
			R: TGAAACCACTTCCGGGAG
Tnip1	m^6A RIP	mouse	F: ATCCTAAGAATGAGGTGCAAC
			R: AACCAAGTCTTTGAGGTTCT
Cre expressioin	Genotyping PCR	mouse	F: CCAGGCTGACCAAGCTGAG
			R: CCTGGCGATCCCTGAACA
Fto flox	Genotyping PCR	mouse	F: AAAGTTTGAAGGAGGGGAGAAGTG
			R: ACCAAAGAGGGGGAGACAGTTACG
$Fto^{\Delta/\Delta}$	Genotyping PCR	mouse	F: CTGAATAGCCCCCTCCCAATGACC
			R: ATAAAAATGACAGGAAGCCAAGAA

PCR: polymerase chain reaction.

Supplemental Table 6. Antibodies and reagents used in this study

Chemicals and Reagents	Catalog Number	Company
Human IL-18 ELISA Kit	ab215539	Abcam
Mouse IL-18BP ELISA Kit	ab254509	Abcam
Human IL-1 beta ELISA Kit	ab214025	Abcam
Mouse IL-1 beta ELISA Kit	ab197742	Abcam
Rabbit monoclonal to FTO	ab280081	Abcam
Anti-YTHDF1 antibody [EPR22349-41]	ab220162	Abcam
Rabbit recombinant multiclonal [RM1006] to CD31	ab281583	Abcam
Mouse monoclonal to beta Actin	ab8226	Abcam
Rabbit Anti-Mouse IgG H&L(HRP)	ab6728	Abcam
Goat Anti-Rabbit IgG H&L(HRP)	ab6721	Abcam
Pierce Magnetic RNA-Protein Pull-Down Kit	20164	Thermo Fisher Scientific
Rhodamine phalloidin	R415	Thermo Fisher Scientific
Imprint® RNA Immunoprecipitation Kit	RIP-12RXN	Sigma-Aldrich
Dual-Glo Luciferase® Assay System	E2920	Promega
GloMax® 96 Microplate Luminometer	E6521	Promega
Rabbit polyclonal antibody to TNIP1	15104-1-AP	Proteintech
Ubiquitin antibody	10201-2-AP	Proteintech
Rabbit polyclonal antibody to HA	51064-2	Proteintech
NF-κB p105/p50 (D4P4D) Rabbit mAb	13586	CST
m^6A Rabbit mAb	D9D9W	CST
DYKDDDDK Tag (D6W5B) Rabbit mAb	14793S	CST
Rabbit monoclonal to A20	5630S	CST
Anti-rabbit IgG, HRP-linked Antibody	7074	CST
Anti-mouse IgG, HRP-linked Antibody	7076	CST
Rabbit monoclonal to FLAG M2	14793	CST

Unedited blot and gel images.

Figure 1E

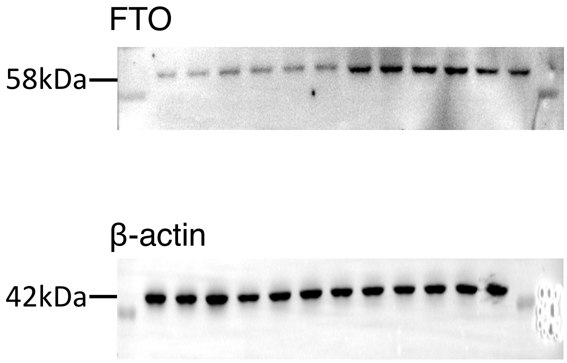

Figure 1F

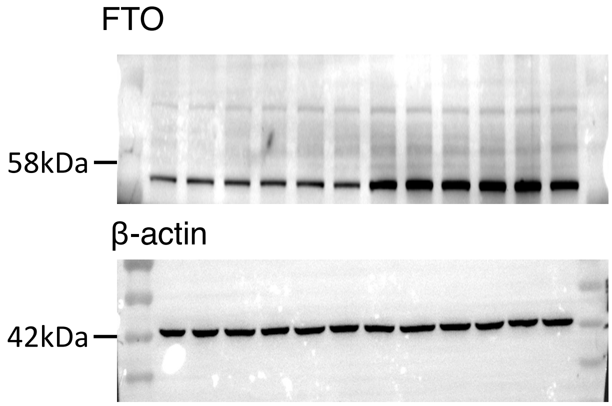

Figure 2D

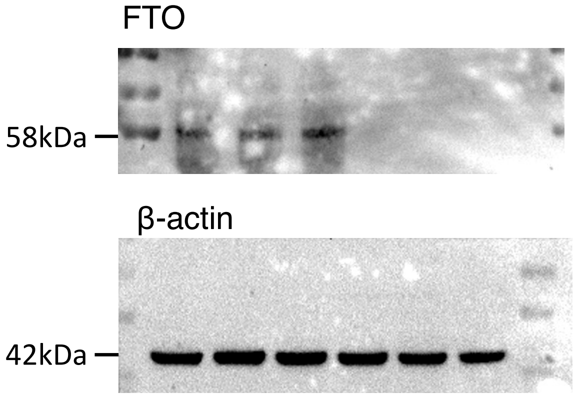

Figure 4A

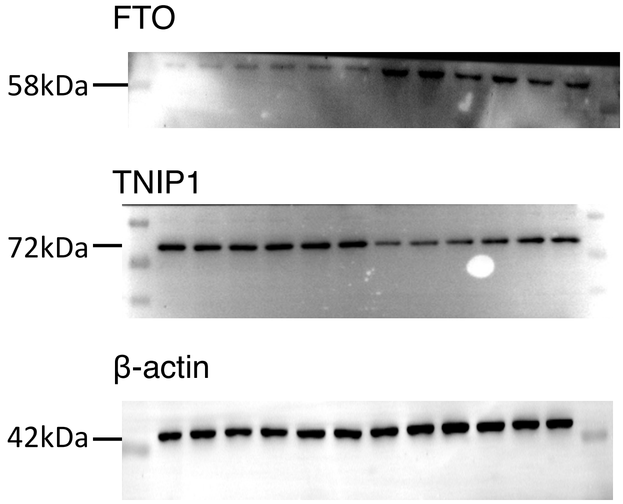

Figure 4B

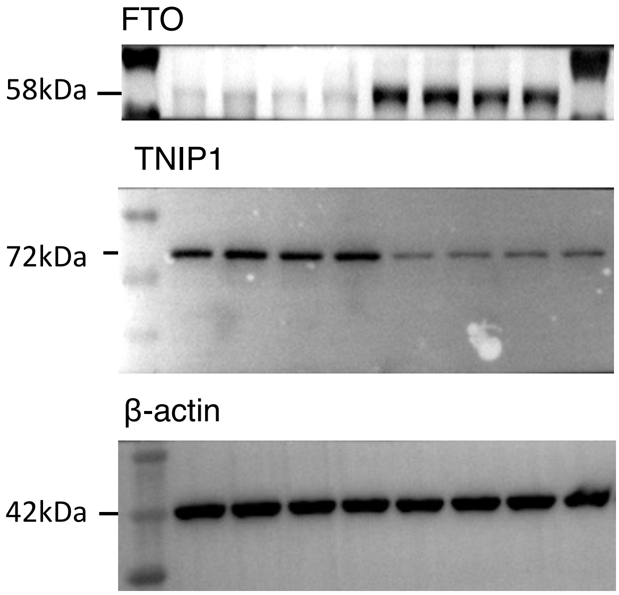

Figure 4C

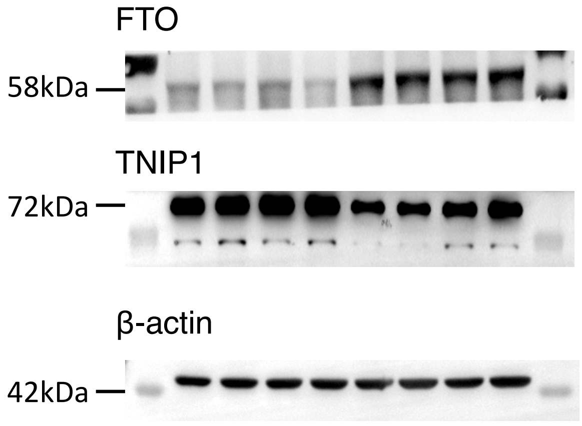

Figure 6A

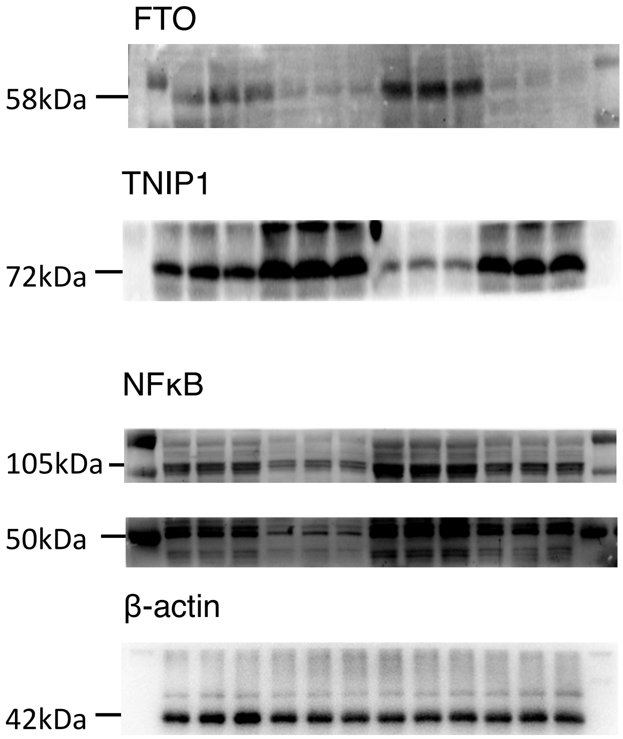

Figure 6B

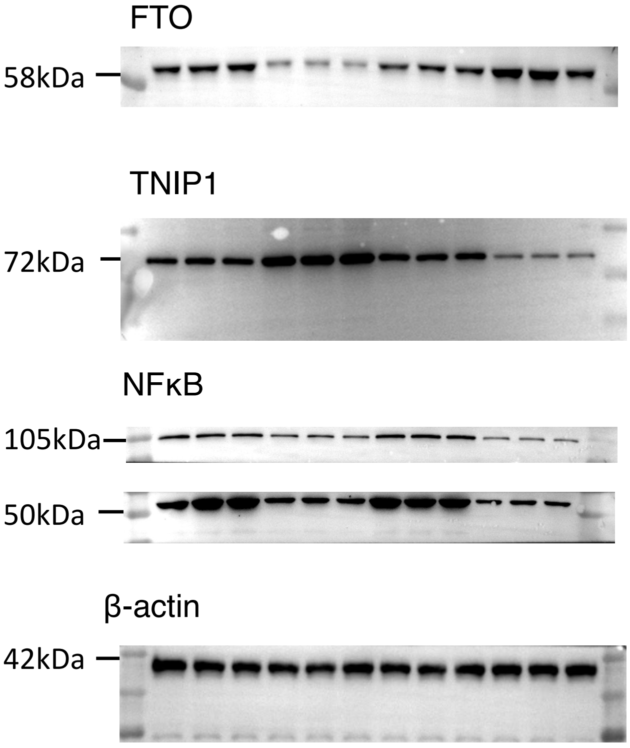

Figure 7F

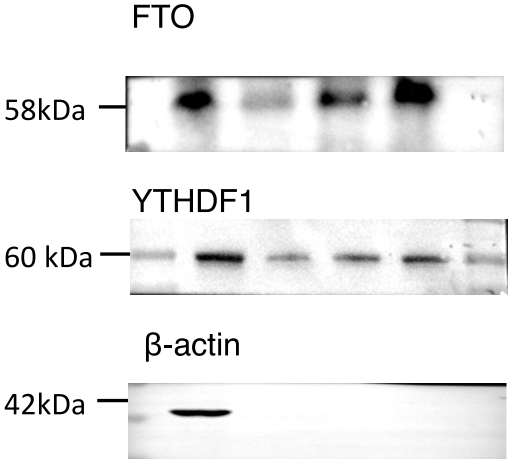

Figure S1F

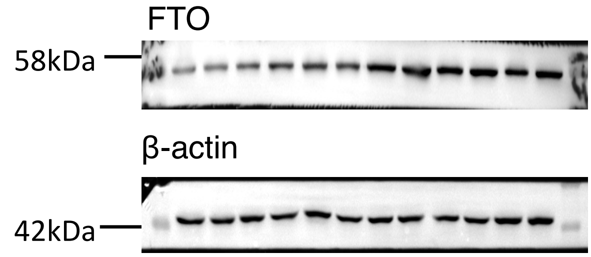

Figure S1G

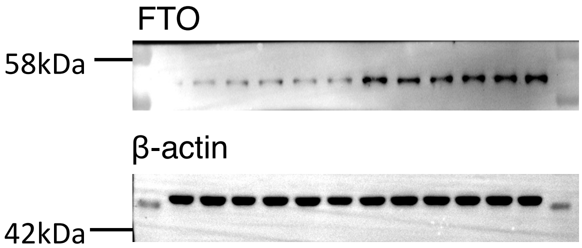

Figure S1H

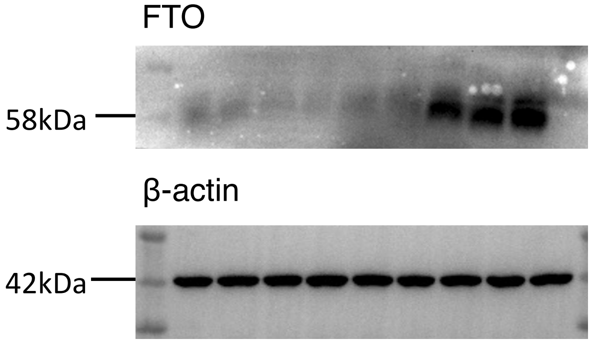

Figure S1I

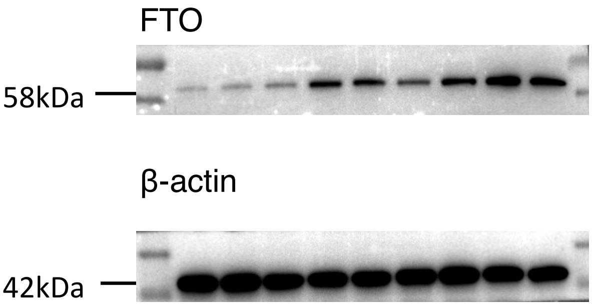

Figure S2D

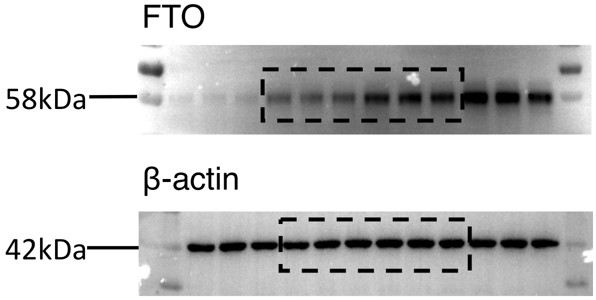

Figure S2E

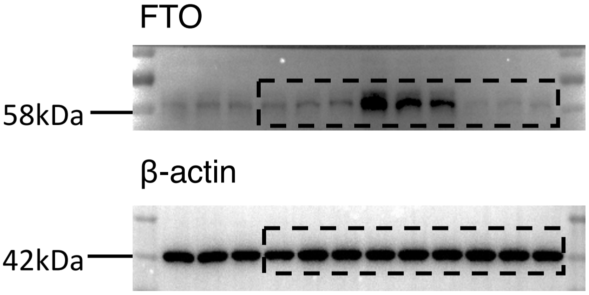

Figure S3A

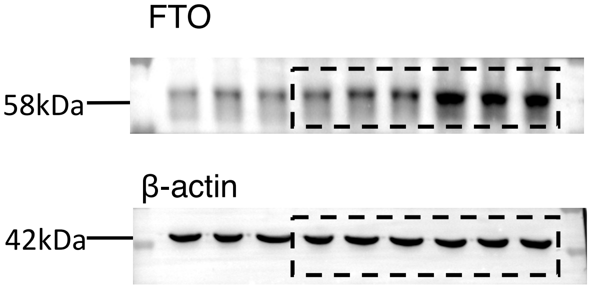

Figure S3B

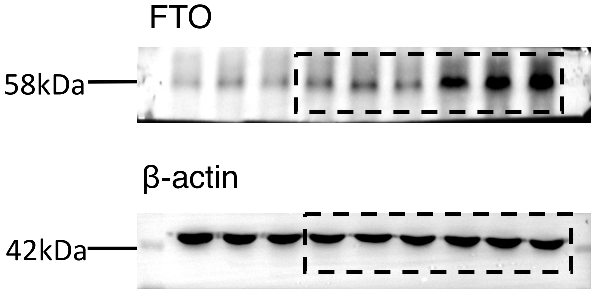

Figure S5A

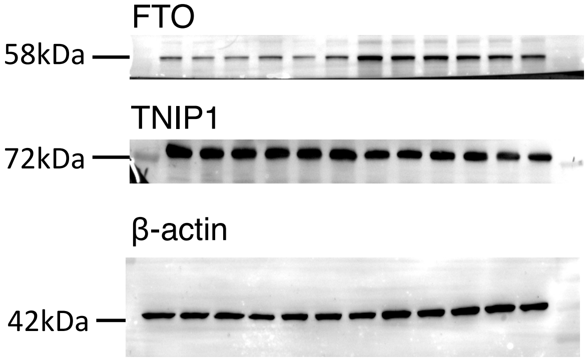

Figure S6G

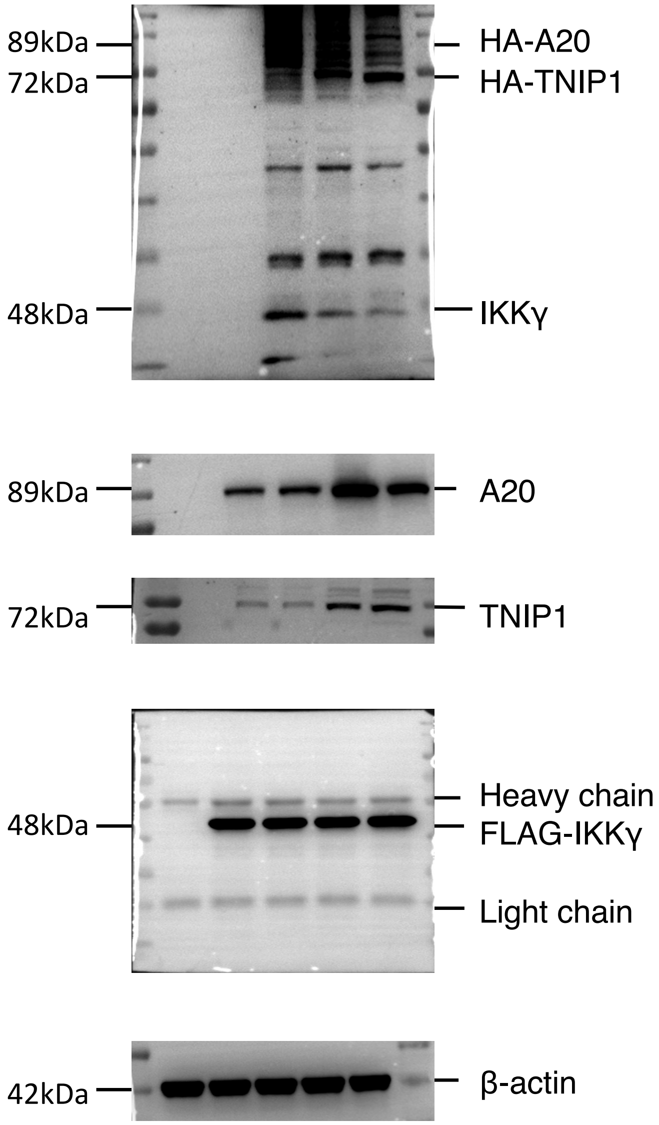

Figure S6E

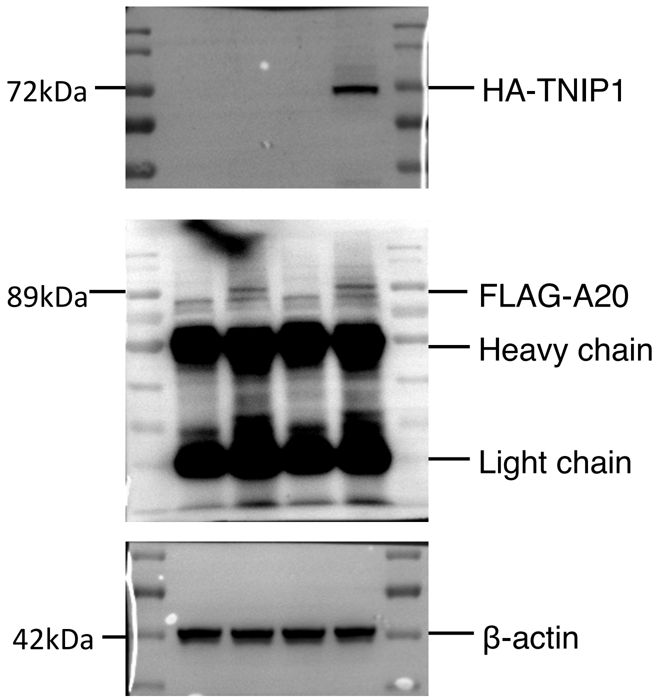

Figure S6F

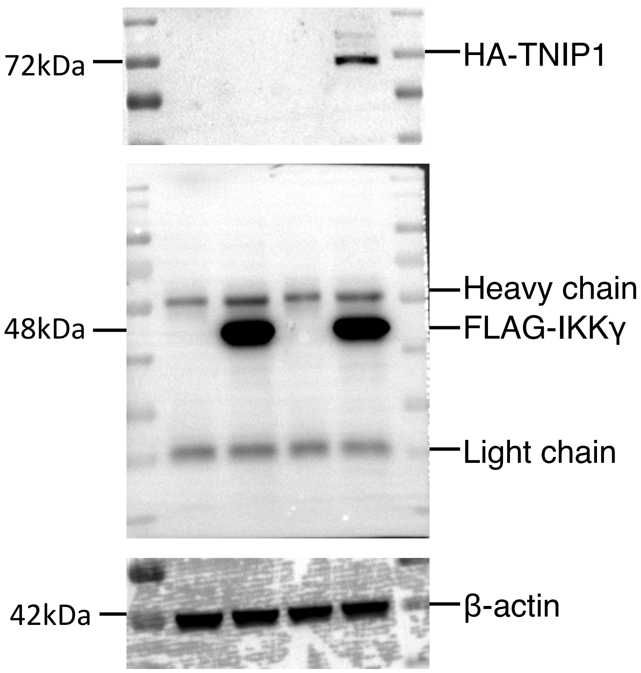

Figure 2C

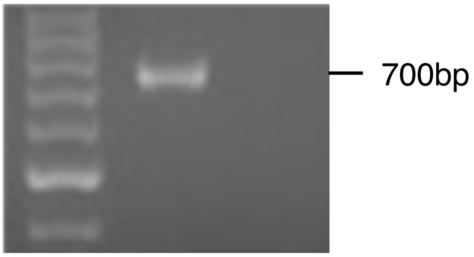

Figure 4D

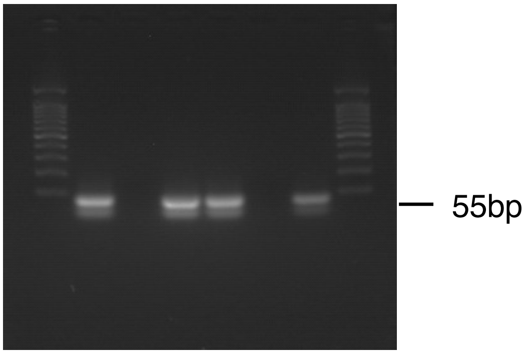

Figure 4E

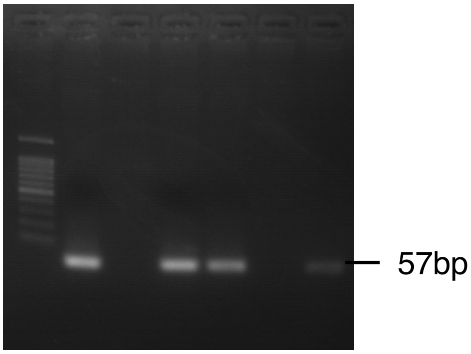

Figure 4F

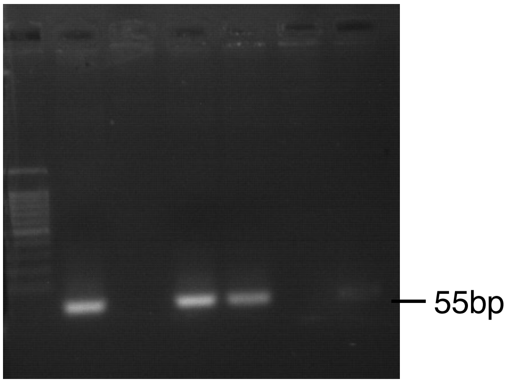

Figure 7A

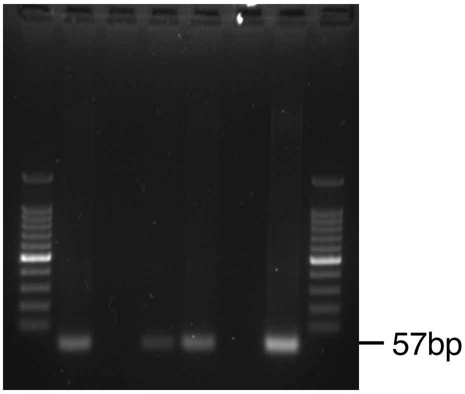

Figure 7B

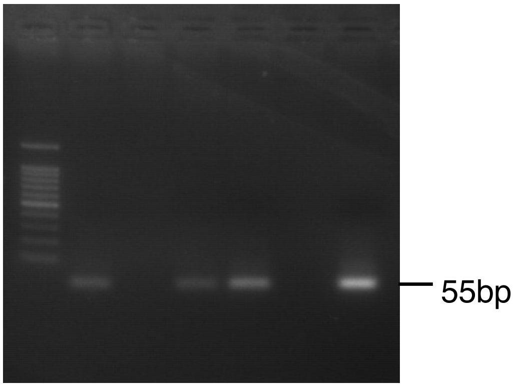

Figure 7F

RNA probes

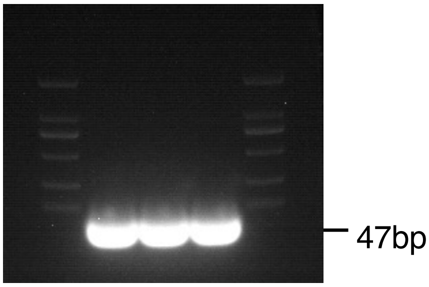

Supplement: Supplemental data [file jci-133-160517-s231.pdf]
